# Locomotion Control Strategy Design and Simulation of Parallel-Legged Insect-Scale Micro Crawling Robot

**DOI:** 10.3390/biomimetics11090603

**Published:** 2026-08-24

**Authors:** Qunwei Zhu, Tao Jiang, Zirong Luo, Yiming Zhu, Guanhai Huang

**Affiliations:** 1 College of Intelligence Science and Technology, National University of Defense Technology, Changsha 410073, China; zhuqunwei@nudt.edu.cn (Q.Z.); jiangtao@nudt.edu.cn (T.J.); yiming.zhu@nudt.edu.cn (Y.Z.); huangguanhai@nudt.edu.cn (G.H.); 2 National Key Laboratory of Equipment State Sensing and Smart Support, National University of Defense Technology, Changsha 410073, China

**Keywords:** insect-scale robot, parallel-legged robot, microrobot control, PID control simulation

## Abstract

High nonlinearity and limited computational resources create persistent challenges for achieving autonomous, stable, and accurate locomotion in insect-scale crawling robots, hindering their practical deployment. In this study, we build the mathematical models of the insect-scale micro crawling robot named PLioBot and propose a locomotion control strategy that eliminates the need for gait transitions. The locomotion transformation of the PLioBot prototype, from driving inputs to mechanical motion outputs, is decomposed into multiple motion processes. This study analyzes the theoretical models underlying these motion processes and develops the locomotion simulation model for the PLioBot based on the mathematical models and the multibody dynamics simulation tool. The locomotion control strategy with low computational requirements is designed based on a closed-loop PID controller, which regulates the robot’s locomotion by independently adjusting the step lengths of its left and right legs. The locomotion co-simulation system is established to validate the control strategy. The simulation results confirm that this control strategy enables the PLioBot to perform straight-line locomotion and turning without requiring any gait transition.

## 1. Introduction

Insect-scale robots are miniature biomimetic robots inspired by the morphology and locomotion principles of insects. Insect-scale micro crawling robots typically refer to robots with a body length of less than 10 cm and mass less than 10 g [1]. Leveraging their miniature size, insect-scale crawling robots can efficiently navigate confined environments such as caves and tunnels [2,3,4] and can be applied in fields such as disaster relief, cargo transportation, and environmental surveying.

Currently, research on insect-scale micro crawling robots primarily focuses on the structural design, actuation design, and robot fabrication [5,6]. For the structural design, the most representative insect-scale robots are the HAMRs (Harvard Ambulatory MicroRobots) developed by the Harvard Microrobotics Laboratory [7,8,9,10,11,12,13]. In addition, recent representative microrobots, including BHMbot [14], Leglessbot [15], and Moobot [16], have demonstrated superior high-speed locomotion capabilities and environmental adaptability. For the actuation design, with the development of smart materials, an increasing number of smart actuators are being investigated and employed in insect-scale microrobots [17]. Currently, the most widely adopted actuator types include piezoelectric ceramic actuators [17,18,19], dielectric elastomer actuators (DEAs) [20], and shape memory alloys (SMAs) [21,22]. For robot fabrication, the most widely adopted approach to date is the Smart Composite Microstructure (SCM) fabrication technique proposed by Harvard University [23,24]. Based on the SCM method, the robot’s actuators, hinges and linkages can be fabricated by stacking, bonding, and curing multiple composite materials [25,26,27,28,29].

However, motion control of integrated insect-scale microrobots remains difficult due to the system’s high nonlinearity and the microcontroller chips’ limited computational capability. Microrobots are typically fabricated from multiple nonlinear composite materials, exhibiting pronounced nonlinear behavior and coupled rigid–flexible dynamics. In the control research of insect-scale micro crawling robots, establishing accurate robot models and constructing reliable simulation environments remain challenging. In addition, insect-scale robots are constrained by limited onboard hardware resources, which hinders the implementation of sophisticated control algorithms. Existing control research for micro crawling robots relies on an external computing device, making onboard implementation on resource-constrained microprocessors challenging for real-time robotic motion control [30,31,32].

**Figure 1:**
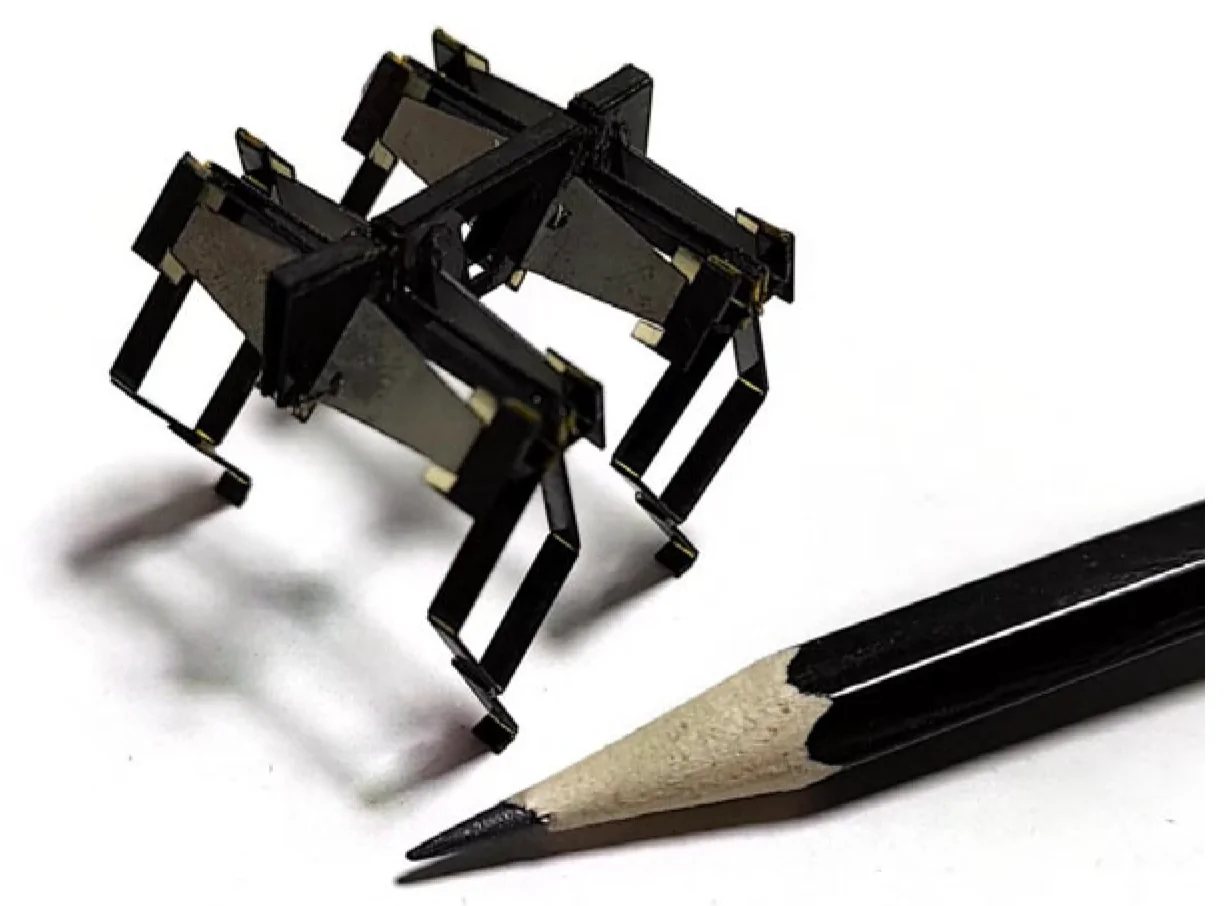
The insect-scale micro crawling robot (PLioBot).

The PLioBot is an insect-scale micro crawling robot, featuring a parallel leg mechanism actuated by piezoelectric actuators, as shown in Figure 1 [19]. This paper conducts the mathematical model analysis, locomotion control strategy design, and locomotion control simulation of the PLioBot. The main innovations are as follows: (1) This study presents a detailed analysis of the locomotion process of the PLioBot and establishes mathematical models for each locomotion process. By integrating the robotic theoretical models with the multibody dynamics simulation tool, the robotic locomotion simulation model is constructed, and its key simulation parameters are systematically determined. (2) We propose a PID-based locomotion control strategy that eliminates the need for gait transitions. The robot operates without switching between gaits or locomotion modes, thereby reducing the complexity of the controller design. Leveraging the robotic simulation model and locomotion control system, the locomotion co-simulation control system is constructed. The simulation results demonstrate that the control method can effectively achieve straight-line movement, fixed-angle turning, in-place turning, and nominal path generation for the PLioBot.

## 2. Materials and Methods

### 2.1. Platform Overview

**Figure 2:**
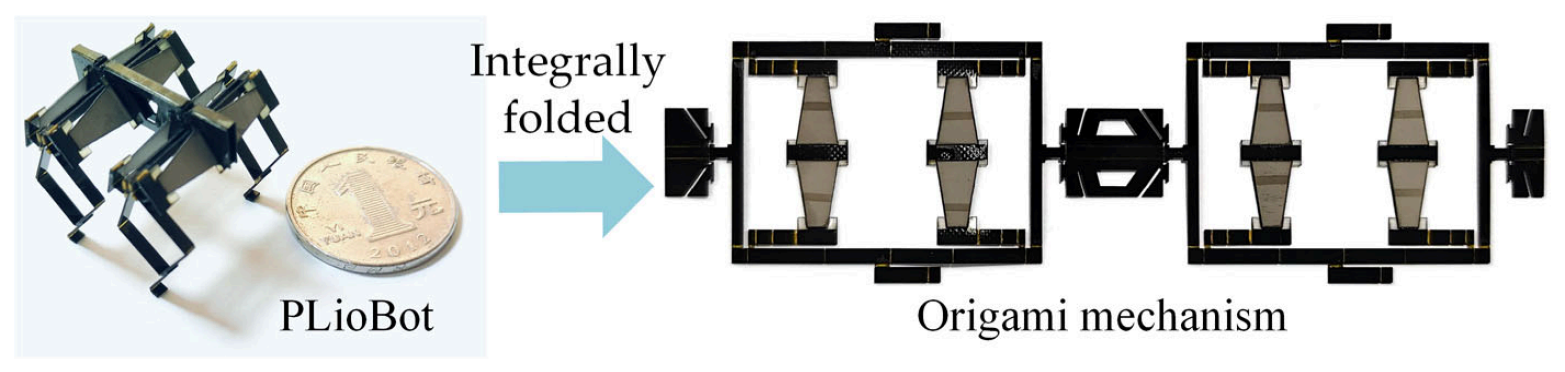
PLioBot and the integrally origami mechanism.

**Figure 3:**
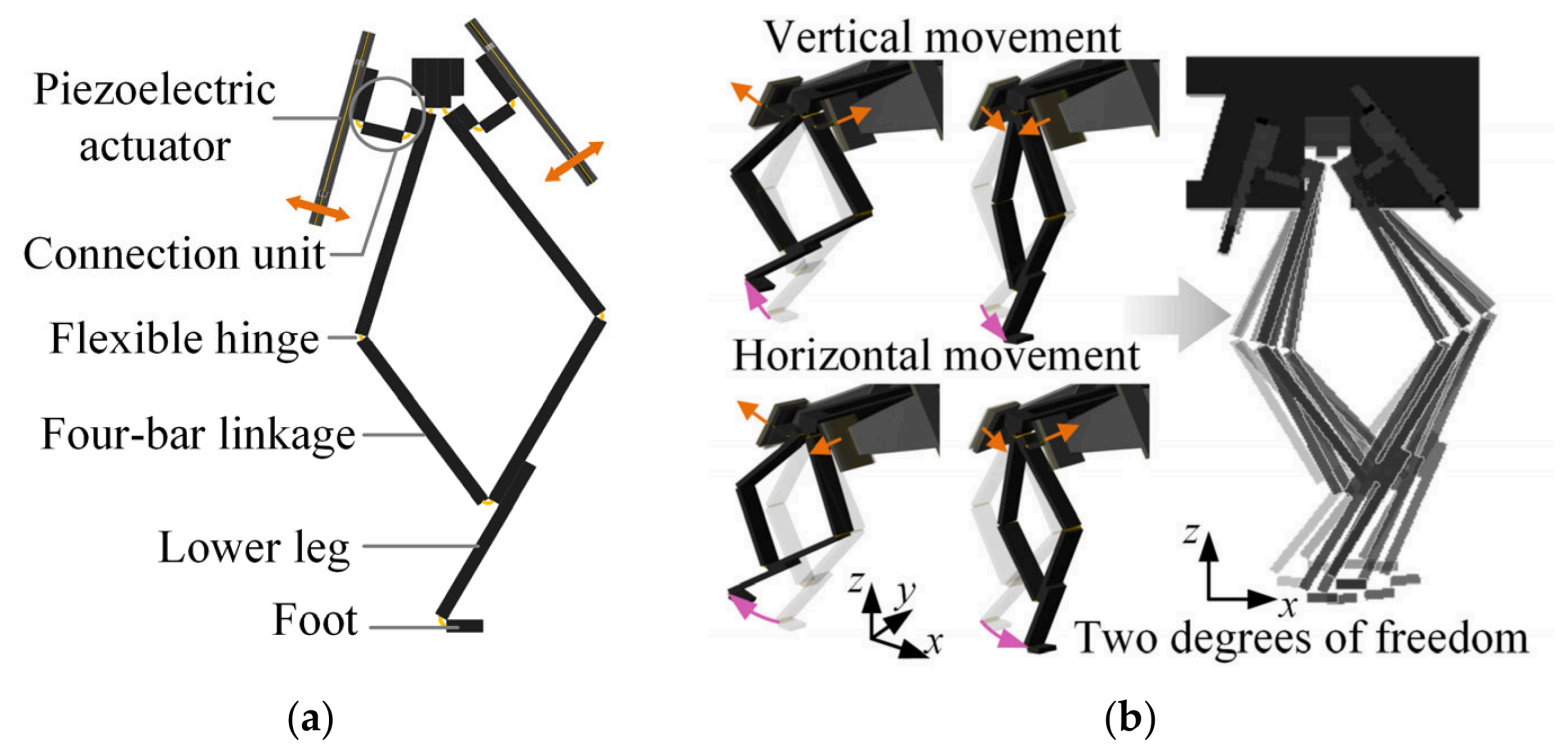
Parallel-legged mechanism and its two-degrees-of-freedom motion. The orange arrows represent the displacement of the piezoelectric actuators, whereas the pink arrows illustrate the parallel-legged locomotion. (**a**) The structural configuration of the parallel-legged mechanism. (**b**) The vertical and horizontal movement of the robot leg.

Earlier, we designed an insect-scale quadrupedal micro crawling robot named PLioBot, which can be integrally folded via an origami mechanism (Figure 2) [19]. PLioBot features a piezoelectrically actuated parallel-legged mechanism, as shown in Figure 3a. The parallel-legged mechanism consists of two piezoelectric actuators, a connection unit, a flexible hinge, a four-bar linkage, a lower leg, and a foot. With the alternating driving signals, the piezoelectric actuator is capable of bidirectional bending. Each leg of the PLioBot is designed to operate with two degrees of freedom, enabling both vertical and horizontal movements, as shown in Figure 3b. The actuator motion is indicated by the yellow arrows, whereas the foot motion is represented by the pink arrows.

**Figure 4:**
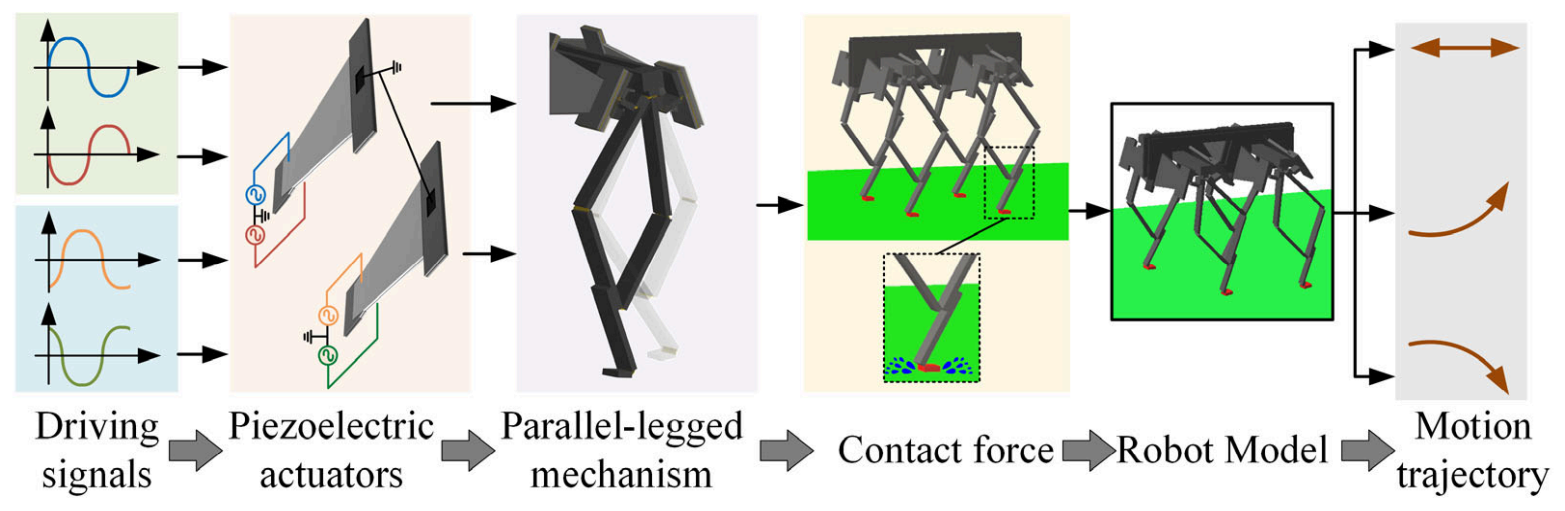
The multiple processes decomposition of the PLioBot’s motion.

The motion of PLioBot can be decomposed into multiple processes, as illustrated in Figure 4. The input to the PLioBot is the driving signals applied to the piezoelectric actuators, while the output is the resulting motion trajectory of the robot system. The motion transmission process from input to output in the robotic system can be broadly divided into four steps. Firstly, the two piezoelectric actuators in the parallel-legged mechanism undergo bending deformation upon application of the driving signals. Secondly, the displacement generated by the piezoelectric actuators serves as the actuation input to the parallel-legged mechanism, enabling two-degrees-of-freedom motion of the robot’s foot. Subsequently, the parallel legs generate the desired foot trajectory and apply contact forces and frictional interactions with the ground during the stance phase. Lastly, the robot achieves straight-line locomotion, turning, and other locomotor behaviors through the synergistic effect of foot actuation and inertial motion.

### 2.2. Mathematical Model

#### 2.2.1. Piezoelectric Actuator Model

**Figure 5:**
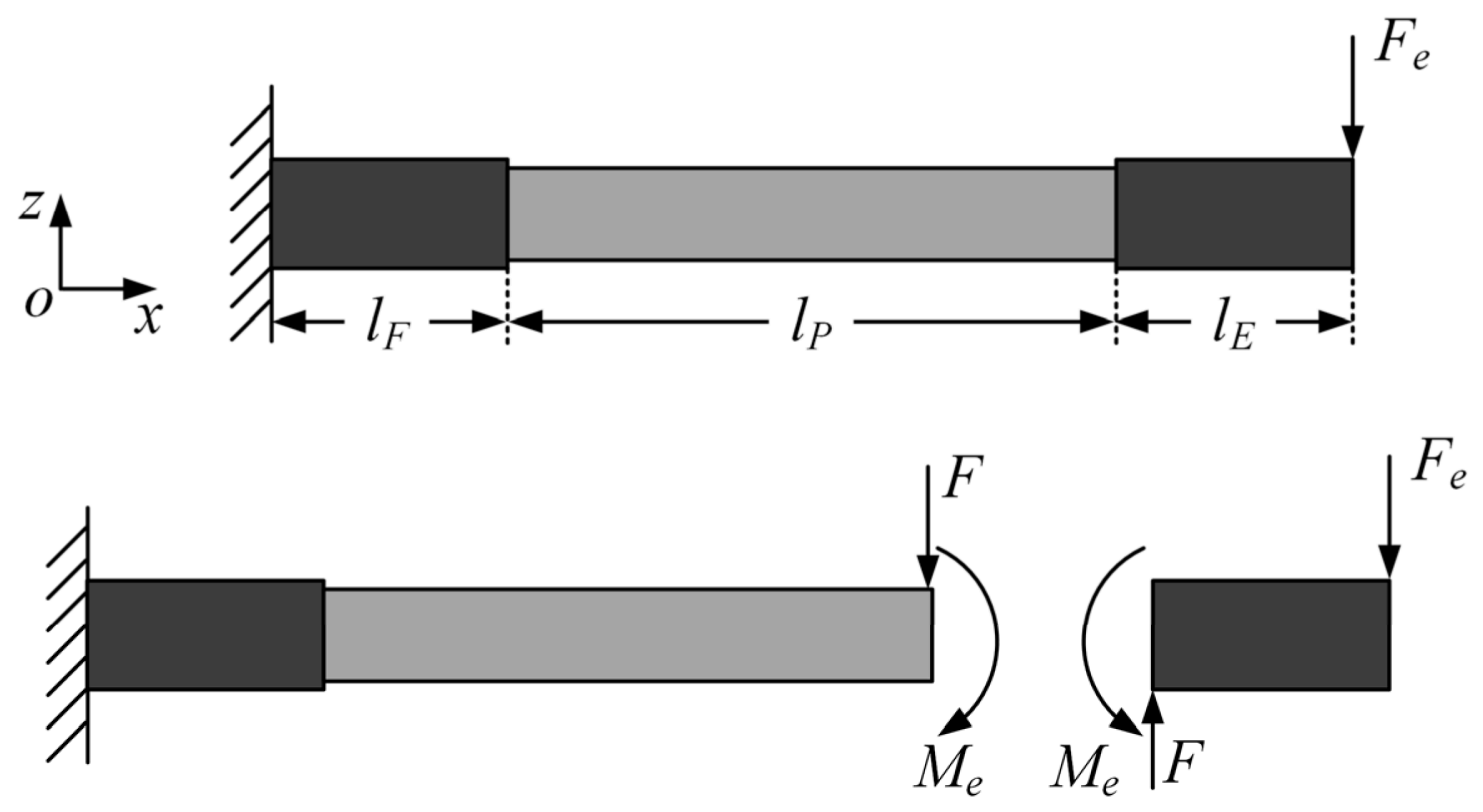
The lateral-view structure of the piezoelectric actuator.

**Figure 6:**
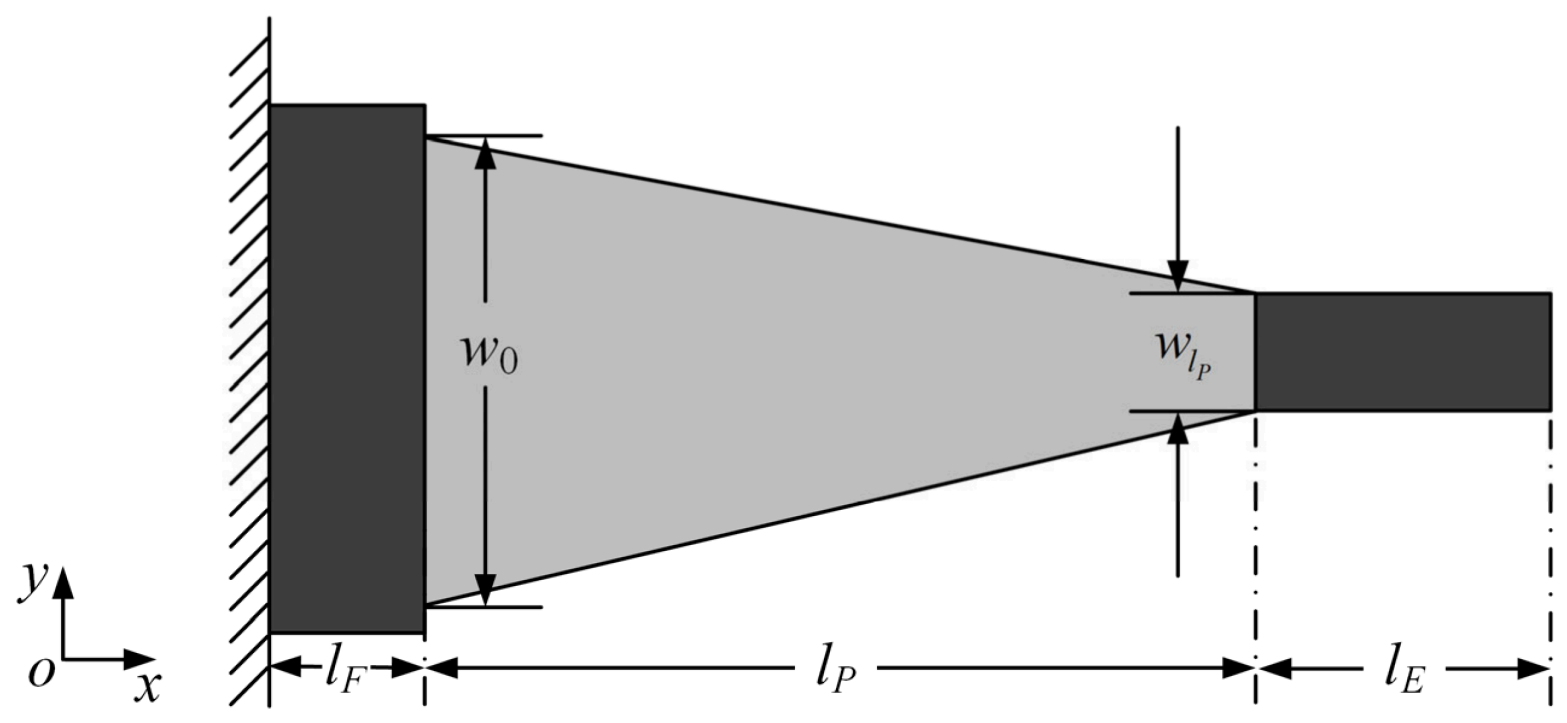
The top-view structure of the piezoelectric actuator.

For the piezoelectric actuator employed in PLioBot, its lateral-view structure is illustrated in Figure 5, while its top-view structural configuration is depicted in Figure 6. The bottom of the piezoelectric actuator is fixed to the frame, while its top end extension segment remains unconstrained and free to move. *F_e_* denotes the external load applied at the tip of the top end extension segment of the piezoelectric actuator. *F* denotes the equivalent external force applied at the piezoelectric ceramic end of the actuator. *M_e_* denotes the torque generated by the external load *F_e_*. *l_F_* denotes the length of the extension segment at the fixed bottom of the piezoelectric actuator. *l_P_* denotes the length of the piezoceramic segment. *l_E_* denotes the length of the extension segment at the top end of the piezoelectric actuator. *w_lP_* and *w*_0_ denote the top width of the piezoelectric actuator and the bottom width at its fixed bottom end, respectively.

According to the elementary beam theory [33], the displacement output of the piezoelectric actuator along the *z*-axis is related to the bending curvature *K_x_*, which can be expressed as

(1) $$\frac{d^{2}\delta (x)}{dx^{2}}=K_{x},$$

where *δ*(*x*) denotes the output displacement of the piezoelectric actuator; *x* denotes its axial length.

Based on the theory of composite material mechanics [33], the bending curvature can be calculated by

(2) $$\frac{d^{2}\delta (x)}{dx^{2}}=P(E_{3})-\frac{F_{e}C_{44}}{w_{\mathrm{nom}}}\frac{\left(l_{P}^{2}(1+l_{r})-l_{P}x\right)}{2(1-w_{r})x+l_{P}w_{r}},$$

where *P*(*E*_3_) denotes the piezoelectric effect function, and *w_nom_*, *w_r_*, and *l_r_* are dimensionless ratio factors, defined as

(3) $$\left\{\begin{array}{l} P(E_{3})=C_{41}N_{x}^{P}+C_{42}N_{y}^{P}+C_{44}M_{x}^{P}+C_{45}M_{y}^{P},\\ w_{nom}=\frac{w_{0}+w_{l_{P}}}{2},\\ w_{r}=\frac{w_{0}}{w_{nom}},\\ l_{r}=\frac{l_{E}}{l_{P}}. \end{array}\right.$$

For *x* = *l_P_*, Equation (2) can be expressed as

(4) $$\delta (l_{P})=\frac{1}{2}P(E_{3})l_{P}^{2}-\frac{C_{44}Fl_{P}^{3}}{w_{nom}}R(w_{r}),$$

where

(5) $$R(w_{r})=-\frac{3-2w_{r}}{4{\left(1-w_{r}\right)}^{2}}+\frac{{\left(2-w_{r}\right)}^{2}}{8{\left(1-w_{r}\right)}^{3}}\ln\frac{2-w_{r}}{w_{r}}.$$

Assuming no external forces are applied, the piezoelectric actuator is influenced by the electric field. Equation (4) can be simplified as

(6) $$\delta (l_{P})=\frac{1}{2}P(E_{3})l_{P}^{2}.$$

#### 2.2.2. Flexure Hinge Model

**Figure 7:**
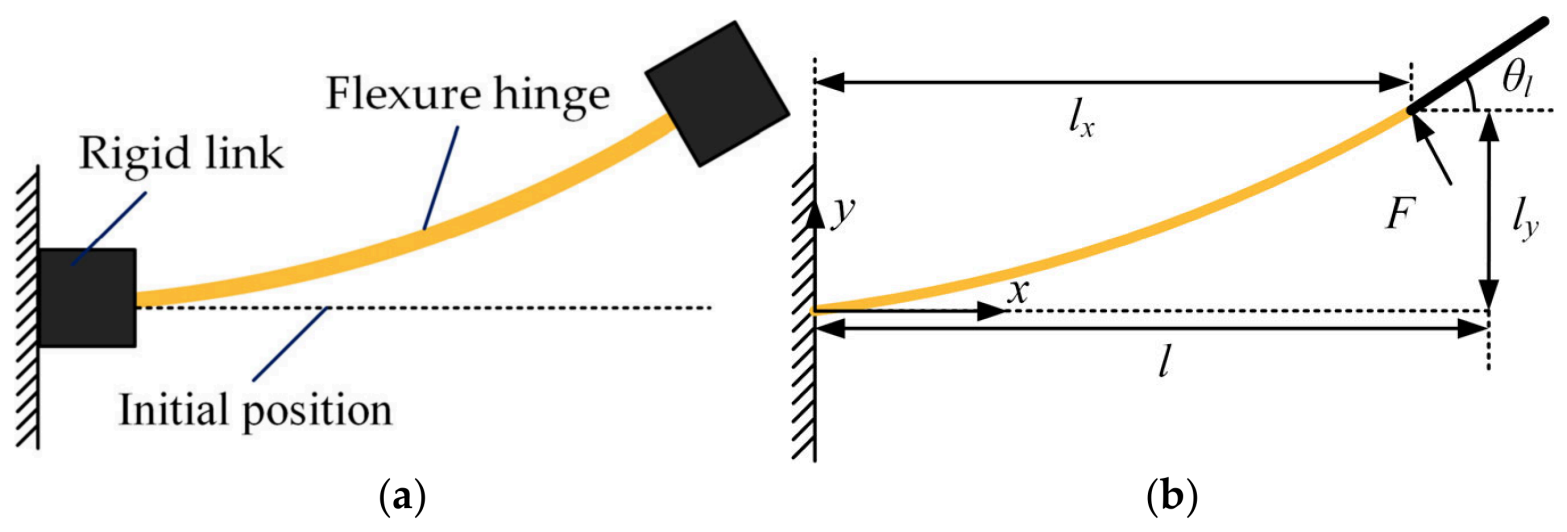
The rigid–flexible coupled structure and its bending deformation. (**a**) The rigid–flexible coupled structure. (**b**) The bending deformation of the hinge.

The rigid–flexible coupled structure is illustrated in Figure 7a and comprises a flexure hinge and two rigid links. One end of the flexure hinge is connected to the fixed rigid link, while the other end remains unconstrained and is free to undergo bending motion. When an external force *F* is applied, the flexure hinge undergoes passive bending, as shown in Figure 7b. The flexure hinge has a length of *l*, a width of *w*, and a thickness of *t_P_*. The rigid link forms the angle *θ_l_* with the horizontal *x*-axis. The displacements of the moving link along the *x*-axis and *y*-axis are denoted by *l_x_* and *l_y_*, respectively.

**Figure 8:**
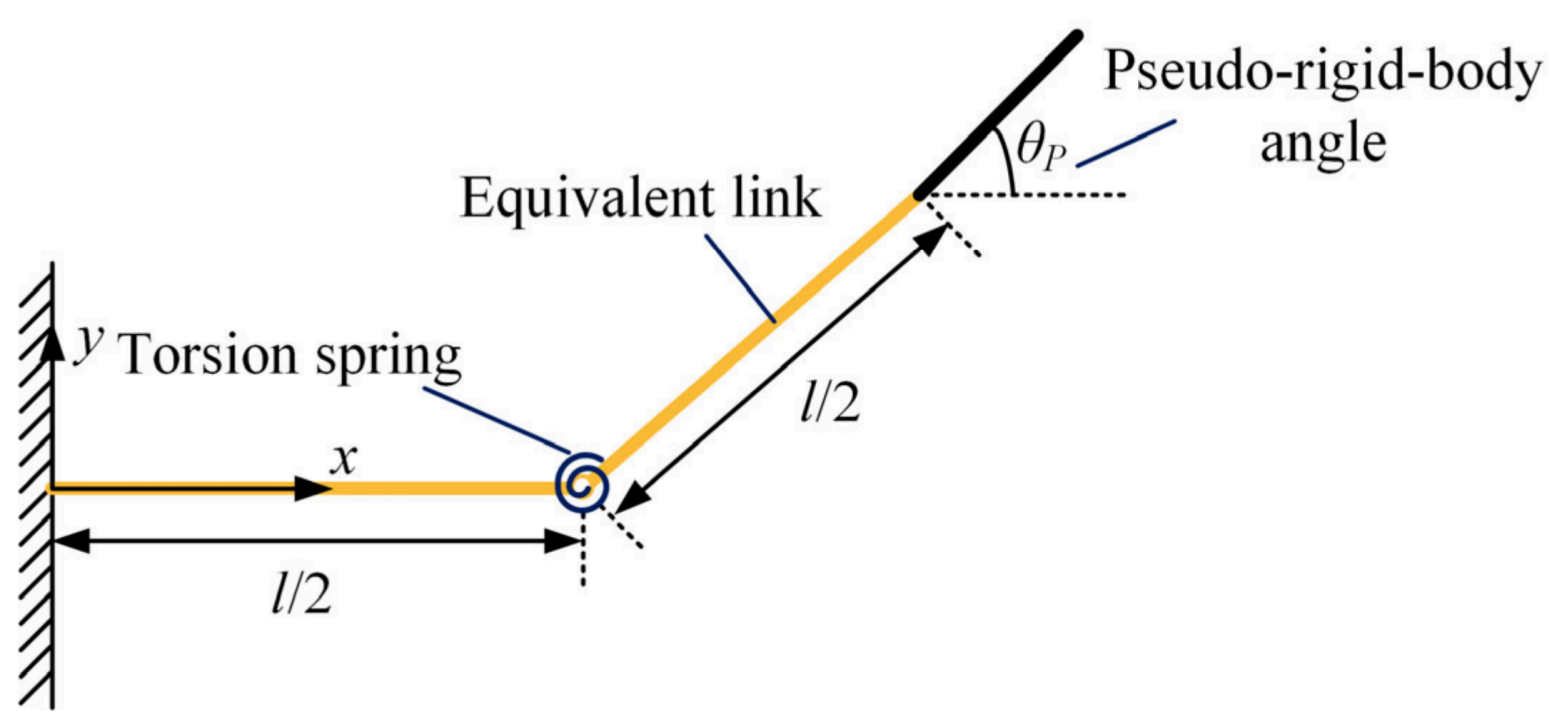
The PRBM of the flexure hinge.

The length of the flexure hinge is significantly smaller than that of the rigid links. According to the pseudo-rigid-body model (PRBM) [34], the bending deformation of the flexible hinge can be approximated as the rotation of two equivalent links about a torsion spring, as shown in Figure 8. The torsion spring is located at the center of the flexure hinge, and the equivalent link length equals half the hinge length. The equivalent link rotates about the torsional spring, generating a pseudo-rigid-body angle *θ_P_*, which can be expressed as

(7) $$\theta_{P}=\theta_{l}=\frac{Ml}{EI},$$

where *M* denotes the externally applied bending torque, *E* is the material’s Young’s modulus, and *I* denotes the second moment of area. *I* can be expressed as

(8) $$I=\frac{wt_{p}^{3}}{12}.$$

The equivalent torsional spring stiffness *k* of the flexure hinge is expressed as

(9) $$k=\frac{EI}{l}.$$

According to the principles of geometry, the deformation displacements *l_x_* and *l_y_* can be expressed as

(10) $$\left\{\begin{array}{l} l_{x}=\frac{l}{2}(1+\cos\theta_{P}),\\ l_{y}=\frac{l}{2}\sin\theta_{P}. \end{array}\right.$$

#### 2.2.3. Parallel-Legged Mechanism Dynamic Model

**Figure 9:**
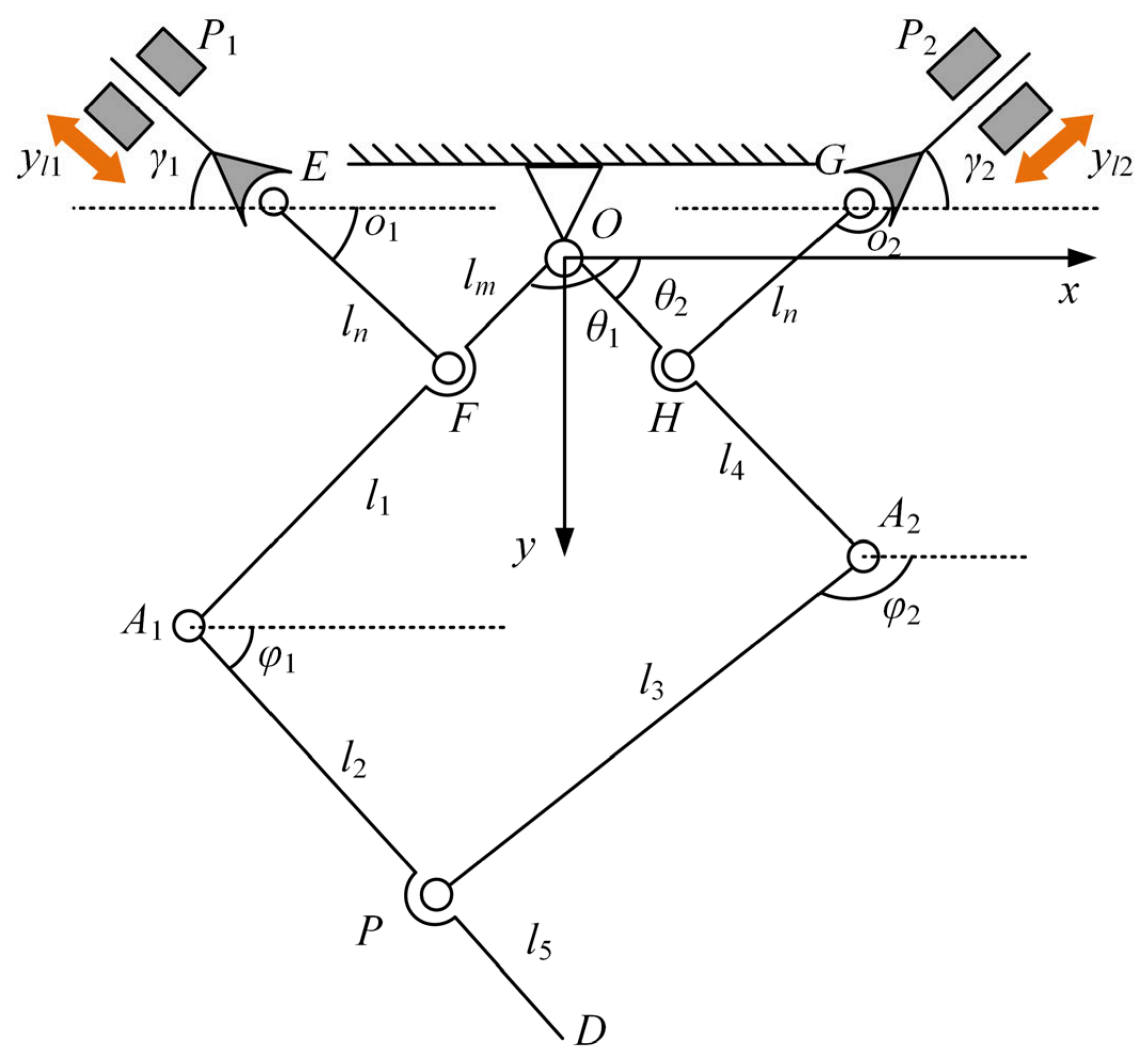
The principle diagram of the parallel-legged mechanism. The dashed lines represent the horizontal direction. The orange arrows represent the displacement of the piezoelectric actuators.

The principle diagram of the parallel-legged mechanism is shown in Figure 9. The points *O*, *A*_1_, *A*_2_, *P*, *F*, *H*, *E*, and *G* represent the flexure hinges, which can be modeled as torsional springs. The two piezoelectric actuators are equivalently modeled as the translational joints *P*_1_ and *P*_2_. The link *A*_1_*P* forms the angle *φ*_1_ with the *x*-axis, and the link *A*_2_*P* makes the angle *φ*_2_ with the *x*-axis. The angles *θ*_1_ and *θ*_2_ denote the angles that the links *OA*_1_ and *OA*_2_ make with the *x*-axis, respectively. The length parameters of all links are listed in Table 1. The displacements of the piezoelectric actuators *P*_1_ and *P*_2_ are denoted by *y_l_*_1_ and *y_l_*_2_, respectively.

The Lagrangian function of the parallel-legged mechanism can be expressed as

(11) $$L(p,\dot{p})=T(p,\dot{p})-U(p),$$

where *T* denotes the total kinetic energy; *U* is the total potential energy. ***p*** is the generalized coordinate vector of the parallel-legged mechanism, which can be defined as

(12) $$\begin{array}{l} p={\left[y_{l1},y_{l2}\right]}^{T},\\ \dot{p}={\left[{\dot{y}}_{l1},{\dot{y}}_{l2}\right]}^{T}. \end{array}$$

The total kinetic energy of the parallel-legged mechanism can be expressed as

(13) $$T=\frac{1}{2}{\dot{p}}^{T}M(p)\dot{p}=\frac{1}{2}{\dot{p}}^{T}(M_{rot}+M_{cm})\dot{p},$$

where ***M*** denotes the mass matrix. ***M****_rot_* and ***M**_cm_* are the mass matrices of piezoelectric actuators and rigid linkages, respectively.

The total potential energy *U* of the parallel-legged mechanism can be expressed as

(14) $$U=U_{g}+U_{s},$$

where *U_g_* denotes the total gravitational potential energy of all components, and *U*_s_ denotes the total elastic potential energy of all flexible hinges. The expressions for *U_g_* and *U*_s_ are

(15) $$\begin{array}{l} U_{g}=-g(m_{l_{1}}y_{C_{OA_{1}}}+m_{l_{4}}y_{C_{OA_{2}}}+m_{left}y_{C_{A_{1}D}}+m_{l_{3}}y_{C_{A_{2}P}})-\\ \;\;\;\;\;\;\;\;\;g(m_{l_{n}}(y_{C_{EF}}+y_{C_{GH}})+m_{P_{1}}(y_{P_{1}}+y_{P_{2}})), \end{array}$$

(16) $$U_{s}=\frac{1}{2}\sum_{i}k_{i}\delta_{i}^{2},$$

where *g* denotes the gravitational acceleration. *m_l_*_1_, *m_l_*_4_, *m_l_*_eft_, *m_l_*_3_, *m_ln_*, and *m_P_*_1_ denote the mass of the link *OA*_1_, *OA*_2_, *A*_1_*D*, *EF*, and piezoelectric actuator *P*_1_, respectively. *k_i_* denotes the equivalent torsional stiffness of the flexure hinge *i*. *δ_i_* denotes the rotation angle of the flexure hinge *i*. *y_Ci_* denotes the height of the center of mass of link *i*, which can be expressed as

(17) $$\left\{\begin{array}{l} y_{C_{OA_{1}}}=\frac{l_{1}}{2}\sin\theta_{1},\\ y_{C_{OA_{2}}}=\frac{l_{4}}{2}\sin\theta_{2},\\ y_{C_{A_{1}D}}=\frac{l_{1}}{2}\sin\theta_{1}+\frac{l_{2}+l_{5}}{2}\sin\varphi_{1},\\ y_{C_{A_{2}P}}=\frac{l_{4}}{2}\sin\theta_{2}+\frac{l_{1}}{2}\sin\theta_{1}+\frac{l_{2}}{2}\sin\varphi_{1},\\ y_{C_{EF}}=\frac{1}{2}\left(\left(l_{n}+y_{l1}\right)\cos(\theta_{1\_0})+l_{m}\sin(\theta_{1\_0})+l_{m}\sin(\theta_{1})\right),\\ y_{C_{GH}}=\frac{1}{2}\left(-\left(l_{n}+y_{l2}\right)\cos(\theta_{2\_0})+l_{m}\sin(\theta_{2\_0})+l_{m}\sin(\theta_{2})\right), \end{array}\right.$$

(18) $$\left\{\begin{array}{l} y_{P_{1}}=\left(l_{n}+y_{l1}\right)\cos(\theta_{1\_0})+l_{m}\sin(\theta_{1\_0}),\\ y_{P_{2}}=-\left(l_{n}+y_{l2}\right)\cos(\theta_{2\_0})+l_{m}\sin(\theta_{2\_0}). \end{array}\right.$$

The dynamic equation of the parallel-legged mechanism is

(19) $$M\ddot{p}+C\dot{p}+\frac{𝜕U_{g}}{𝜕p}+K(p)=\tau +J_{D}^{T}F_{D},$$

where ***K***(***p***) denotes the nonlinear elastic force term of the flexure hinge, and

(20) $$K(p)=\frac{𝜕U_{s}}{𝜕p}={\left[\frac{𝜕U_{s}}{𝜕y_{l_{1}}},\frac{𝜕U_{s}}{𝜕y_{l_{2}}}\right]}^{T},$$

(21) $$C\dot{p}=\dot{M}\dot{p}-\frac{𝜕T}{𝜕p},$$

(22) $$\dot{M}=\frac{𝜕M}{𝜕\theta_{1}}\frac{𝜕\theta_{1}}{𝜕y_{l_{1}}}{\dot{y}}_{l_{1}}+\frac{𝜕M}{𝜕\theta_{2}}\frac{𝜕\theta_{2}}{𝜕y_{l_{2}}}{\dot{y}}_{l_{2}},$$

(23) $$\left\{\begin{array}{l} C_{11}=\frac{1}{2}(\frac{𝜕M_{11}}{𝜕\theta_{1}}\frac{𝜕\theta_{1}}{𝜕y_{l_{1}}}{\dot{y}}_{l_{1}}+\frac{𝜕M_{11}}{𝜕\theta_{2}}\frac{𝜕\theta_{2}}{𝜕y_{l_{2}}}{\dot{y}}_{l_{2}}),\\ C_{12}=\frac{1}{2}\frac{𝜕M_{11}}{𝜕\theta_{2}}\frac{𝜕\theta_{1}}{𝜕y_{l_{1}}}{\dot{y}}_{l_{1}}+(\frac{𝜕M_{12}}{𝜕\theta_{2}}-\frac{1}{2}\frac{𝜕M_{22}}{𝜕\theta_{1}}\frac{𝜕\theta_{2}}{𝜕y_{l_{2}}}){\dot{y}}_{l_{2}},\\ C_{21}=(\frac{𝜕M_{12}}{𝜕\theta_{1}}-\frac{1}{2}\frac{𝜕M_{11}}{𝜕\theta_{2}})\frac{𝜕\theta_{1}}{𝜕y_{l_{1}}}{\dot{y}}_{l_{1}}+\frac{1}{2}\frac{𝜕M_{22}}{𝜕\theta_{1}}\frac{𝜕\theta_{2}}{𝜕y_{l_{2}}}{\dot{y}}_{l_{2}},\\ C_{22}=\frac{1}{2}(\frac{𝜕M_{22}}{𝜕\theta_{1}}\frac{𝜕\theta_{1}}{𝜕y_{l_{1}}}{\dot{y}}_{l_{1}}+\frac{𝜕M_{22}}{𝜕\theta_{2}}\frac{𝜕\theta_{2}}{𝜕y_{l_{2}}}{\dot{y}}_{l_{2}}). \end{array}\right.$$

#### 2.2.4. Foot–Ground Contact Model

**Figure 10:**
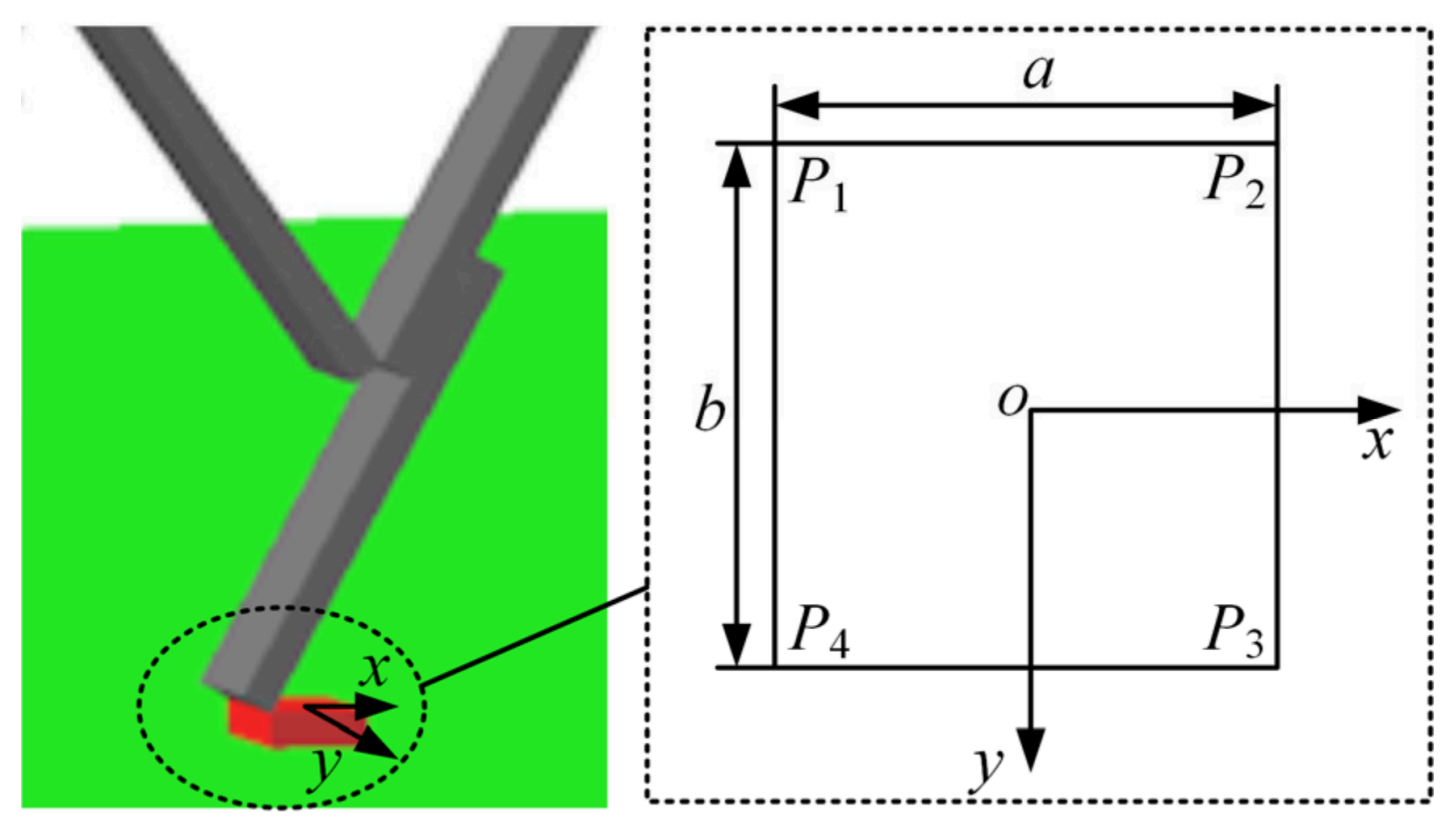
The foot–ground contact model of the PLioBot. The green region denotes the ground. The red square represents the robot’s foot.

The robotic foot is composed of multiple layers of composite materials [23]. The minor deformations of both the ground and the robot’s feet during contact are neglected. For a rectangular foot with length *a* and width *b*, a coordinate system is established with its origin at the geometric center, as illustrated in Figure 10. The four vertices *P*_1_, *P*_2_, *P*_3_, and *P*_4_ of the foot can be expressed as

(24) $$\left\{\begin{array}{l} P_{1}=(-\frac{a}{2},-\frac{b}{2}),\\ P_{2}=(\frac{a}{2},-\frac{b}{2}),\\ P_{3}=(\frac{a}{2},\frac{b}{2}),\\ P_{4}=(-\frac{a}{2},\frac{b}{2}). \end{array}\right.$$

The foot pitch angle is denoted by *α*, representing rotation about the *y*-axis. The foot roll angle is denoted by *β*, representing rotation about the *x*-axis. For *α* > 0, the anterior edge of the foot is lifted, causing points *P*_2_ and *P*_3_ to lose contact with the ground. Conversely, when *α* < 0, the posterior edge of the foot is lifted, causing points *P*_1_ and *P*_4_ to become airborne. For *β* > 0, the right lateral edge of the foot is elevated, resulting in *P*_3_ and *P*_4_ losing contact with the ground. Conversely, for *β* < 0, the left lateral edge is lifted, causing *P*_1_ and *P*_2_ to become airborne.

The world-frame *z*-coordinate of the foot center is denoted by *z_c_*. The *z*-coordinates of the four foot vertices in the world coordinate system can be expressed as

(25) $$\left\{\begin{array}{l} P_{1}:z_{1}=z_{c}-\frac{a}{2}\sin\alpha -\frac{b}{2}\sin\beta ,\\ P_{2}:z_{2}=z_{c}+\frac{a}{2}\sin\alpha -\frac{b}{2}\sin\beta ,\\ P_{3}:z_{3}=z_{c}+\frac{a}{2}\sin\alpha +\frac{b}{2}\sin\beta ,\\ P_{4}:z_{4}=z_{c}-\frac{a}{2}\sin\alpha +\frac{b}{2}\sin\beta . \end{array}\right.$$

Based on the contact area between the foot and the ground, the foot–ground interaction during leg movement can be divided into multiple distinct phases. At the initial contact phase, the foot first contacts the ground, with the plantar surface making point contact. At this instant, only one of the four vertices of the foot’s plantar surface has a *z*-coordinate equal to zero. Both the foot pitch angle *α* and roll angle *β* are nonzero. Assuming that the foot point *P*_1_ makes initial contact with the ground, then

(26) $$\left\{\begin{array}{l} z_{1}=0,\\ z_{2},z_{3},z_{4}>0. \end{array}\right.$$

As the foot continues descending, it eventually makes linear contact with the ground. Depending on the leg posture angle, this linear contact can be categorized into two distinct cases. The first scenario involves line contact between the foot and the ground along the *y*-direction, where the roll angle *β* = 0 and the pitch angle *α* ≠ 0. For instance, consider the line contact along the edge *P*_1_*P*_4_ at the rear of the foot. The *z*-coordinate of the four foot vertices are

(27) $$\left\{\begin{array}{l} z_{1}=z_{4}=0,\\ z_{2},z_{3}>0. \end{array}\right.$$

Substituting *β* = 0 into Equation (25) yields

(28) $$\left\{\begin{array}{l} z_{1}=z_{4}=z_{c}-\frac{a}{2}\sin\alpha ,\\ z_{2}=z_{3}=z_{c}+\frac{a}{2}\sin\alpha . \end{array}\right.$$

The second scenario involves the initial line contact along the *x*-direction at the foot. In this case, the roll angle *β* ≠ 0, while the pitch angle *α* = 0. Taking the left-side line contact between points *P*_1_ and *P*_2_ as an example, the corresponding expressions can be obtained as

(29) $$z_{c}=\frac{b}{2}\sin\beta .$$

As the foot continues to descend, the contralateral edge of the foot gradually approaches the ground until the entire plantar surface makes full contact with the ground. At this instant, the roll angle *β* = 0, and the pitch angle *α* = 0. The foot–ground contact transitions from line contact to surface contact, with the contact area equaling the product of the foot’s length and width. The transition from line contact to surface contact can be approximated as instantaneous. The foot’s contact area jumps directly from zero to the full foot area, with no intermediate variation in contact area.

As the foot lifts off the ground gradually, the contact between the plantar surface and the ground transitions sequentially from surface contact to line contact and then to point contact, ultimately achieving complete detachment.

The contact force between the foot and ground can be decomposed into a normal component and a tangential component. According to the nonlinear Hunt–Crossley model, the contact force *F_c_* is expressed as

(30) $$F_{c}=F_{n}\cdot n+F_{t}\cdot t,$$

(31) $$\left\{\begin{array}{l} F_{n}=k\cdot \delta^{e}+b\cdot \dot{\delta },\\ F_{t}=-\mu F_{n}\cdot \mathrm{sgn}(v_{x}), \end{array}\right.$$

where *F_n_* denotes the normal contact force, *F_t_* denotes the tangential contact force, *δ* is the penetration depth, *k* is the contact stiffness, *e* is the force index, *b* is the damping coefficient, *μ* is the coefficient of friction, and *v_x_* denotes the tangential foot velocity.

During the initial point contact phase, the plantar surface first makes contact with the ground. Consequently, the normal force increases gradually from zero, and its expression is

(32) $$\left\{\begin{array}{l} F_{n}=b\cdot \dot{\delta },\\ F_{t}=-\mu F_{n}\cdot \mathrm{sgn}(v_{x}), \end{array}\right.$$

For the line contact phase during foot touchdown, the maximum penetration depth approximately occurs at the midpoint of the contact edge on the plantar surface. Taking edge contact along the *y*-axis as an example, *δ*_max_ denotes this maximum penetration depth, which can be expressed as

(33) $$\delta_{\max}=\frac{a}{2}\sin\alpha .$$

At this moment, the contact force is defined as the integral over the contact boundary, and its expression is

(34) $$\left\{\begin{array}{l} F_{n}=\int_{-\frac{b}{2}}^{\frac{b}{2}}\bar{k}\cdot b\cdot \delta^{e}(y)dy,\\ \bar{k}=\frac{k}{ab}, \end{array}\right.$$

where $\bar{k}$ denotes the contact stiffness per unit area. The unit of $\bar{k}$ is N/cm^(e+2)^.

If the penetration depth is uniformly distributed, then

(35) $$F_{n}={\left(\frac{a}{2}\sin\alpha \right)}^{e}\bar{k}b^{2}.$$

During the foot–ground surface contact phase, the penetration depth remains constant at *δ*_0_, corresponding to the maximum contact force, which is

(36) $$F_{n}=\bar{k}\delta_{0}^{e}ab.$$

#### 2.2.5. Differential-Stride Turning Locomotion Model

**Figure 11:**
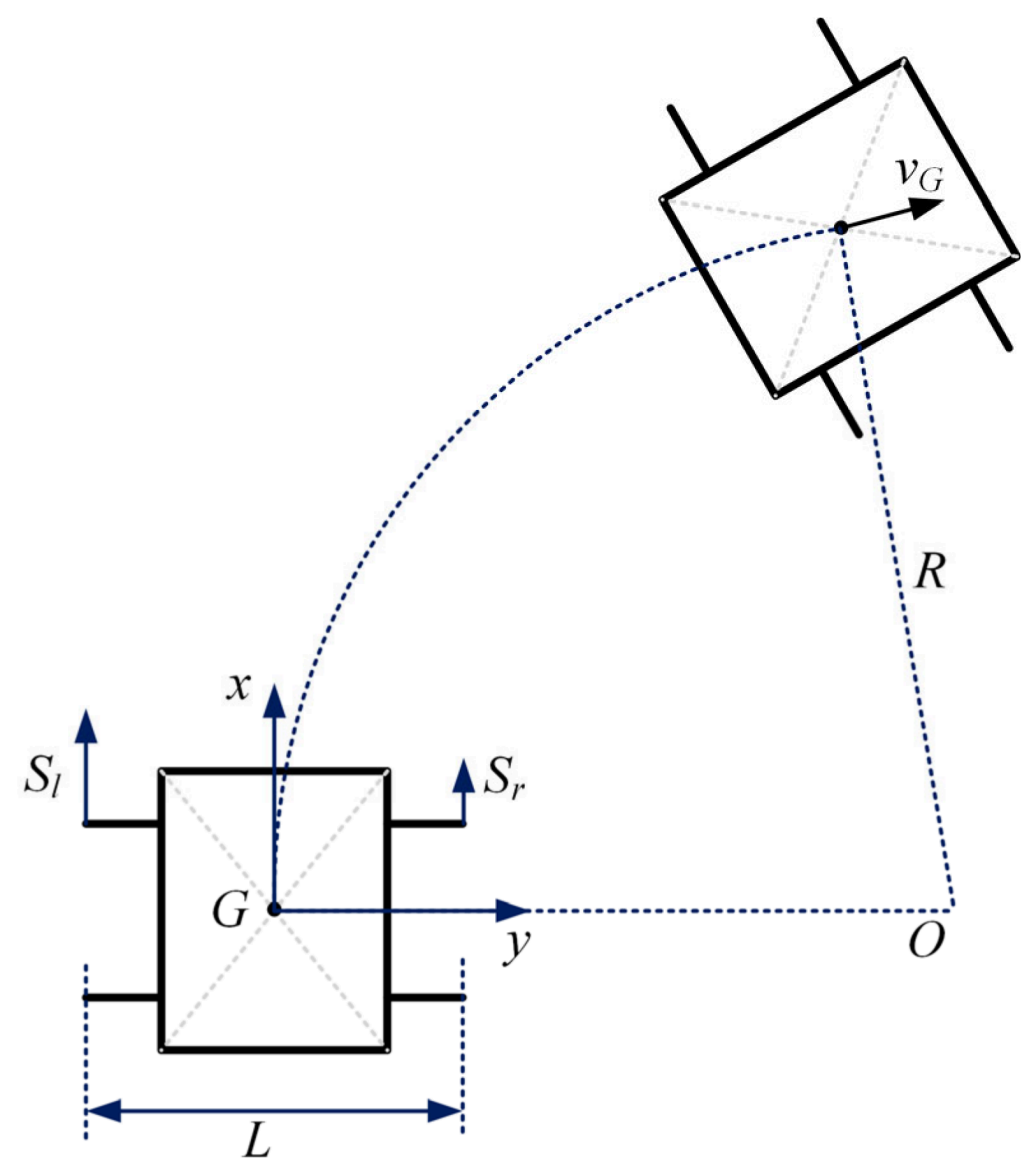
The differential-stride turning locomotion of the PLioBot.

Employing differential step lengths between the left and right legs is the most fundamental turning strategy for quadrupedal robots, enabling the robot to traverse an arc-shaped path [35]. The center of mass of the robot body is assumed to be located at its geometric center, denoted as point *G*. A coordinate system is established with *G* as the origin, as shown in Figure 11, where the forward direction is defined as the positive *x*-axis and the rightward horizontal direction as the positive *y*-axis. The distance between the left and right legs is denoted as *L*, the center of rotation as *O*, the turning radius as *R*, the angular velocity as *ω*, and the linear velocity of the center of mass as *v_G_*. The step lengths of the left and right legs are denoted as *S_l_* and *S_r_*, respectively, and the step period is denoted as *T*. *S_l_* and *S_r_* are signed parameters, with the sign denoting the direction of the leg step. The average forward velocities of the left and right legs relative to the robot body, denoted as *v_l_* and *v_r_*, are then given by:

(37) $$v_{l}=\frac{2S_{l}}{T}$$

(38) $$v_{r}=\frac{2S_{r}}{T}$$

According to rigid-body kinematics, when the velocities of the left and right sides of the robot differ, the robot turns about an instantaneous center of rotation. The turning angular velocity ω and the linear velocity *v_G_* of the center of mass are respectively given by:

(39) $$w=\frac{v_{r}-v_{l}}{L}$$

(40) $$v_{G}=\frac{v_{l}+v_{r}}{2}$$

The turning radius *R* can be expressed as

(41) $$R=\frac{v_{G}}{w}=\frac{L}{2}\frac{v_{r}+v_{l}}{v_{r}-v_{l}}$$

Based on Equations (37) and (38), Equation (41) can be expressed as

(42) $$\left\{\begin{array}{l} R=\frac{L}{2}\frac{S_{r}+S_{l}}{\Delta S}\\ \Delta S=S_{r}-S_{l} \end{array}\right.$$

where Δ*S* represents the difference in step length between the left and right legs. Defining *k_S_* as the step length ratio between the two sides, Equation (42) can be expressed as

(43) $$R=\frac{L}{2}\frac{k_{S}+1}{k_{S}-1}$$

**Figure 12:**
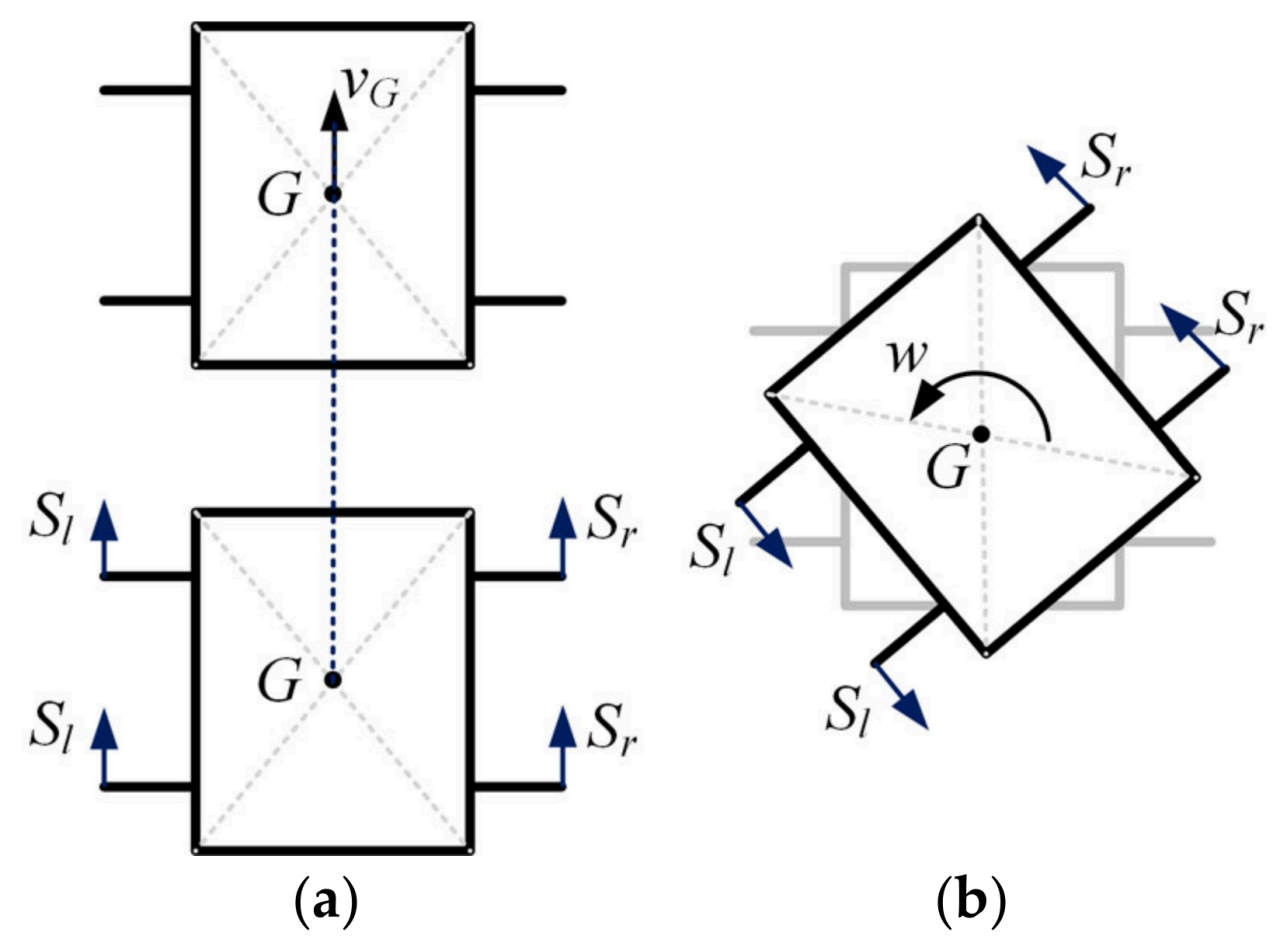
The straight-line locomotion and the in-place turning principle of the robot. (**a**) The straight-line locomotion. (**b**) The in-place turning locomotion.

Based on differential steering kinematics, Δ*S* > 0 implies a larger right-side step length than that of the left side, and *k_S_* > 1, which results in a left turn of the robot. When Δ*S* < 0, the right step length is smaller than the left step length (*k_S_* < 1), resulting in a right turn. In the special case where Δ*S* = 0, the left and right step lengths are equal (*k_S_* = 1), the turning radius approaches infinity, and the robot travels in a straight line, as depicted in Figure 12a. When the step lengths on both sides are equal in magnitude but opposite in sign, *k_S_* = −1, and the turning radius reduces to zero, enabling the robot to perform in-place rotation (Figure 12b). The steering behavior of the robot is summarized in Table 2.

### 2.3. Control Strategy

#### 2.3.1. Locomotion Gait and Foot Trajectory

**Figure 13:**
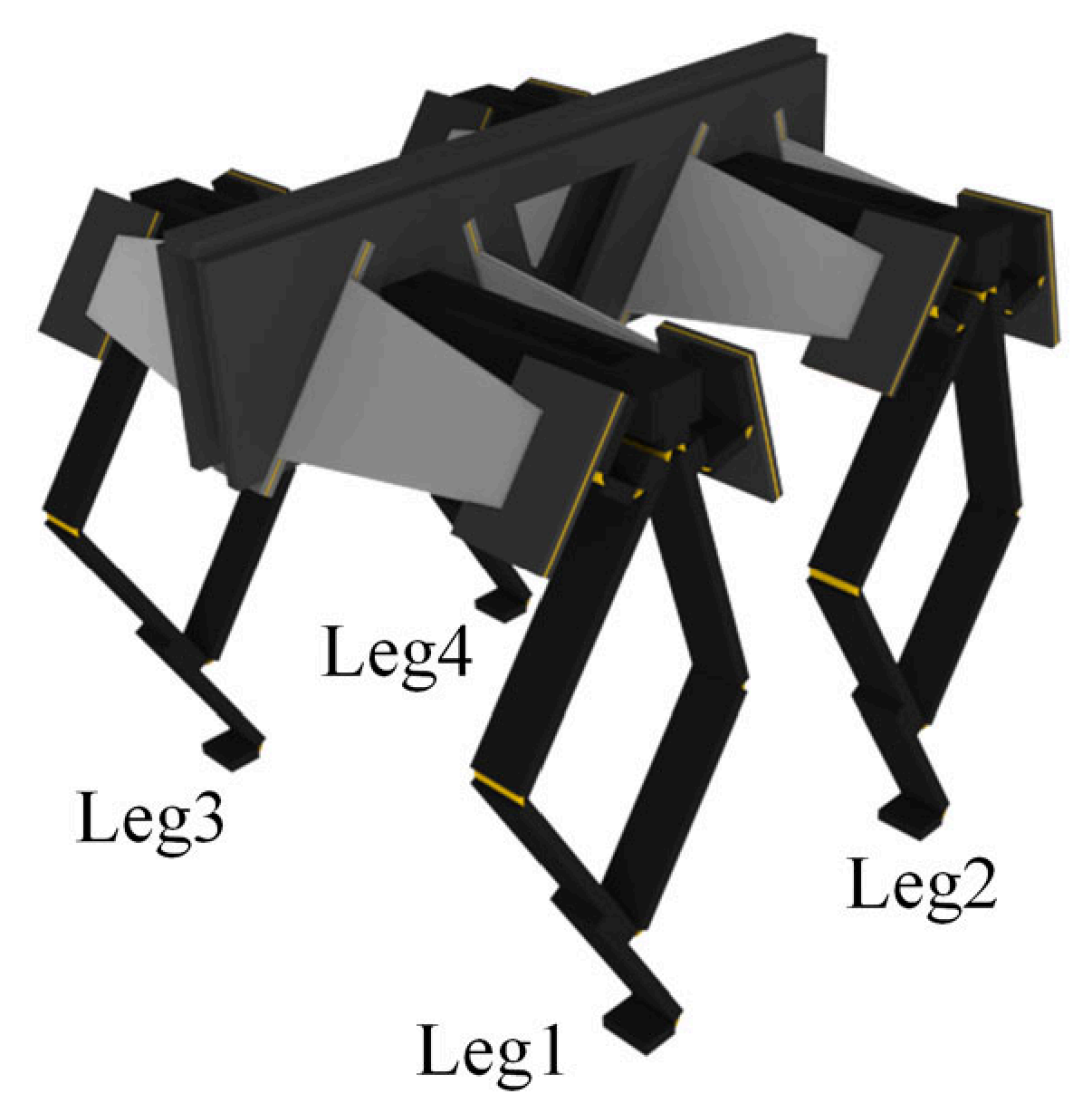
The quadruped number of the PLioBot.

The four legs of the PLioBot are labeled Leg 1, Leg 2, Leg 3, and Leg 4, respectively, as illustrated in Figure 13. Each leg of the PLioBot requires four driving signals, and the four legs collectively require sixteen driving signals. The four legs are grouped into two pairs, with each pair sharing four driving signals; the robot requires only eight driving signals in total.

**Figure 14:**
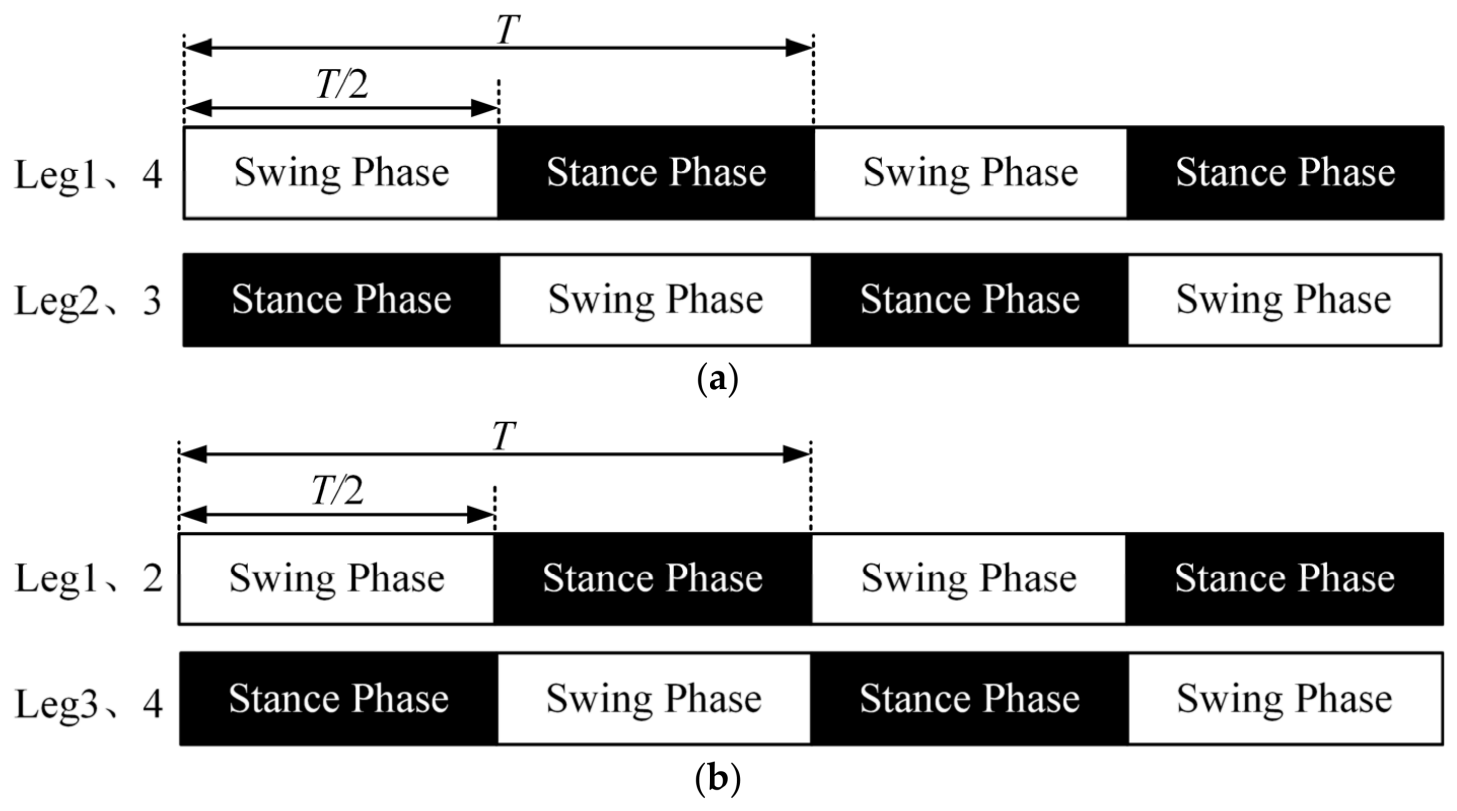
The temporal gait patterns of the robot’s trot and pace gaits (**a**) The trot gait. (**b**) The pace gait.

When the robot’s diagonally opposite legs are paired, with Legs 1 and 4 forming one group and Legs 2 and 3 forming the other, the two legs within each group move in synchrony, and the robot adopts a trot gait. Conversely, when the legs on the same side are paired, with Legs 1 and 2 forming one group and Legs 3 and 4 forming the other, the legs in each group execute identical motion patterns, resulting in a pace gait. The temporal gait patterns of the trot and pace gaits are shown in Figure 14a and b, respectively.

**Figure 15:**
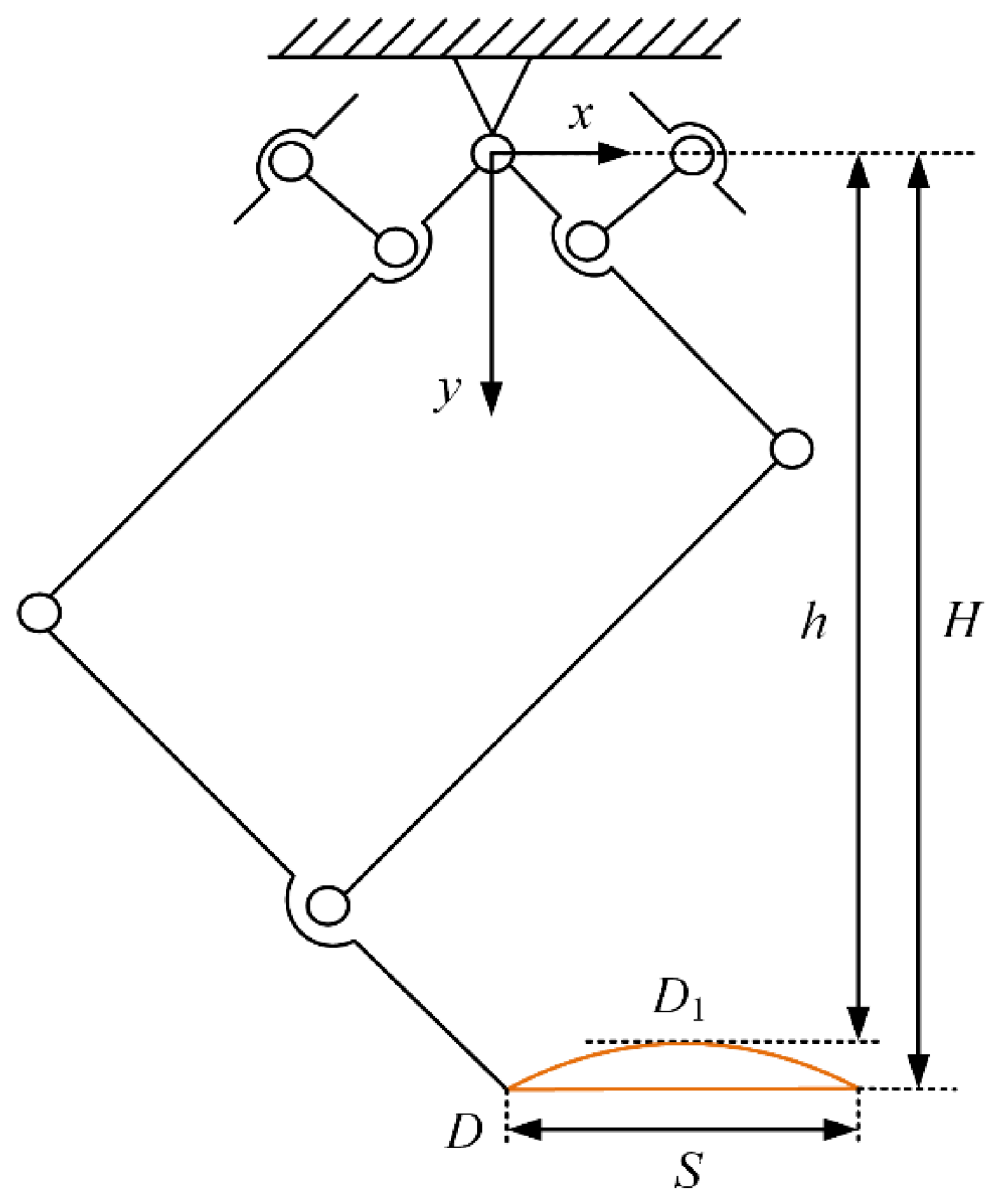
The foot trajectory model of the parallel leg. The orange line denotes the foot trajectory.

Referring to the existing foot trajectory generation methods for quadruped robots, the primary approaches include composite cycloidal trajectory and quintic polynomial bionic trajectory [36]. The initial position of the foot is denoted as point *D*, and the highest leg-lifting position is denoted as point *D*_1_, as illustrated in Figure 15. *S* represents the maximum step-forward length of the foot. *H* denotes the *y*-coordinate of point *D*. *h* denotes the *y*-coordinate of point *D*_1_.

The composite cycloidal trajectory can be expressed as

(44) $$\begin{array}{l} x=\left\{\begin{array}{c} S(\frac{2t}{T}-\frac{1}{2\pi }\sin(\frac{4\pi t}{T}))\;\;\;\;\;\;\;\;\;\;\;\;\;\;\;\;\;\;(0\leq t\leq \frac{T}{2})\\ -S(\frac{2t}{T}-\frac{1}{2\pi }\sin(\frac{4\pi t}{T}))+2S\;\;\;\;\;\;\;\;(\frac{T}{2}\leq t\leq T) \end{array}\right.,\\ y=\left\{\begin{array}{c} 2(H-h)(\frac{2t}{T}-\frac{1}{4\pi }\sin(\frac{8\pi t}{T}))-H\;\;\;\;\;\;\;\;\;\;\;\;\;\;\;\;\;\;(0\leq t\leq \frac{T}{4})\\ \begin{array}{l} 2(h-H)(\frac{2t}{T}-\frac{1}{4\pi }\sin(\frac{8\pi t}{T}))+H-2h\;\;\;\;\;\;\;\;(\frac{T}{4}\leq t\leq \frac{T}{2})\\ -H\;\;\;\;\;\;\;\;\;\;\;\;\;\;\;\;\;\;\;\;\;\;\;\;\;\;\;\;\;\;\;\;\;\;\;\;\;\;\;\;\;\;\;\;\;\;\;\;\;\;\;\;\;\;\;\;\;\;\;\;\;\;\;\;\;\;\;\;\;(\frac{T}{2}\leq t\leq T) \end{array} \end{array}\right.. \end{array}$$

**Figure 16:**
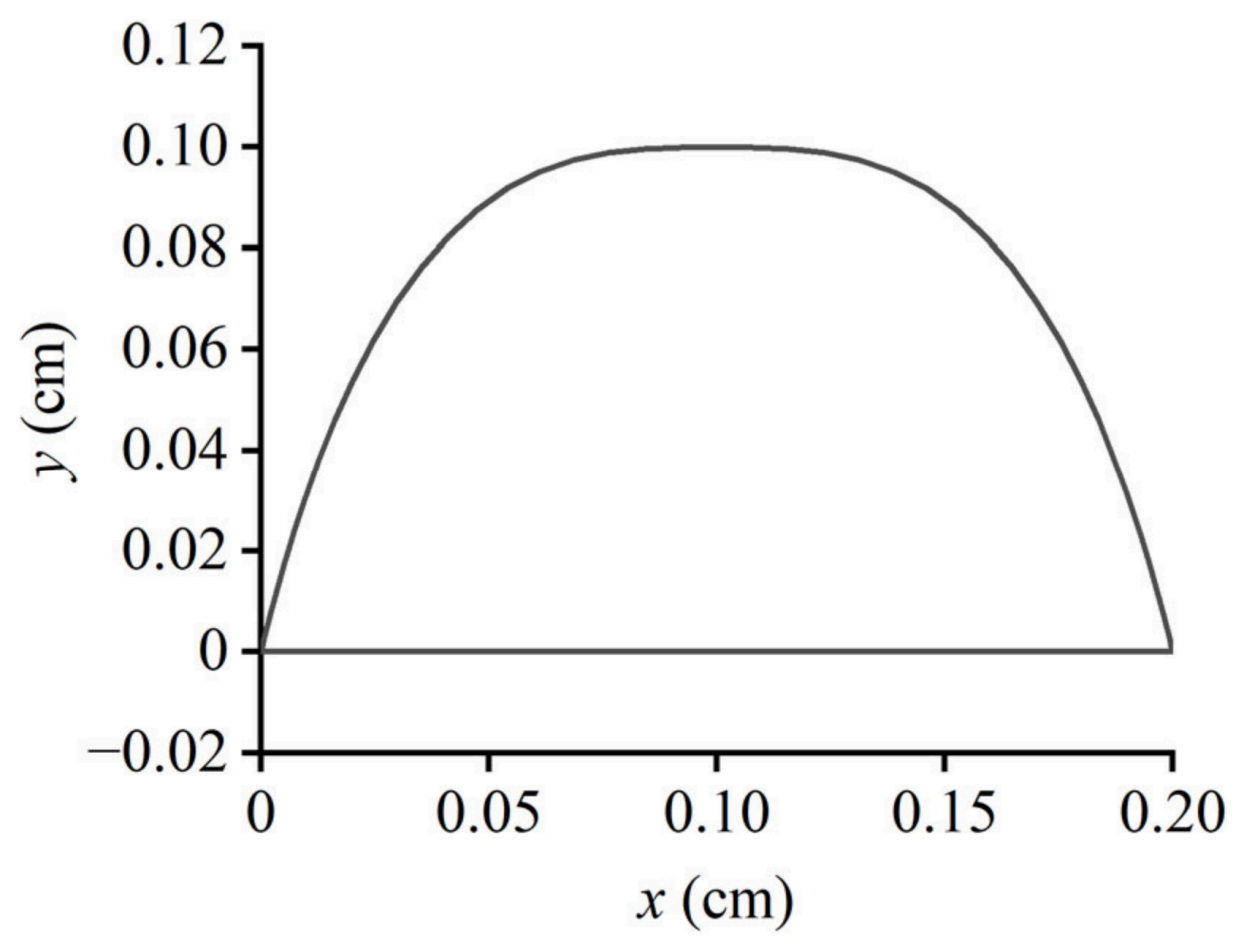
The desired composite cycloidal trajectory.

The PLioBot achieves a leg lift height of 1 mm and a step length of 2 mm. Based on the robot’s kinematic model [19], the resulting composite cycloidal trajectory over one gait cycle is shown in Figure 16.

**Figure 17:**
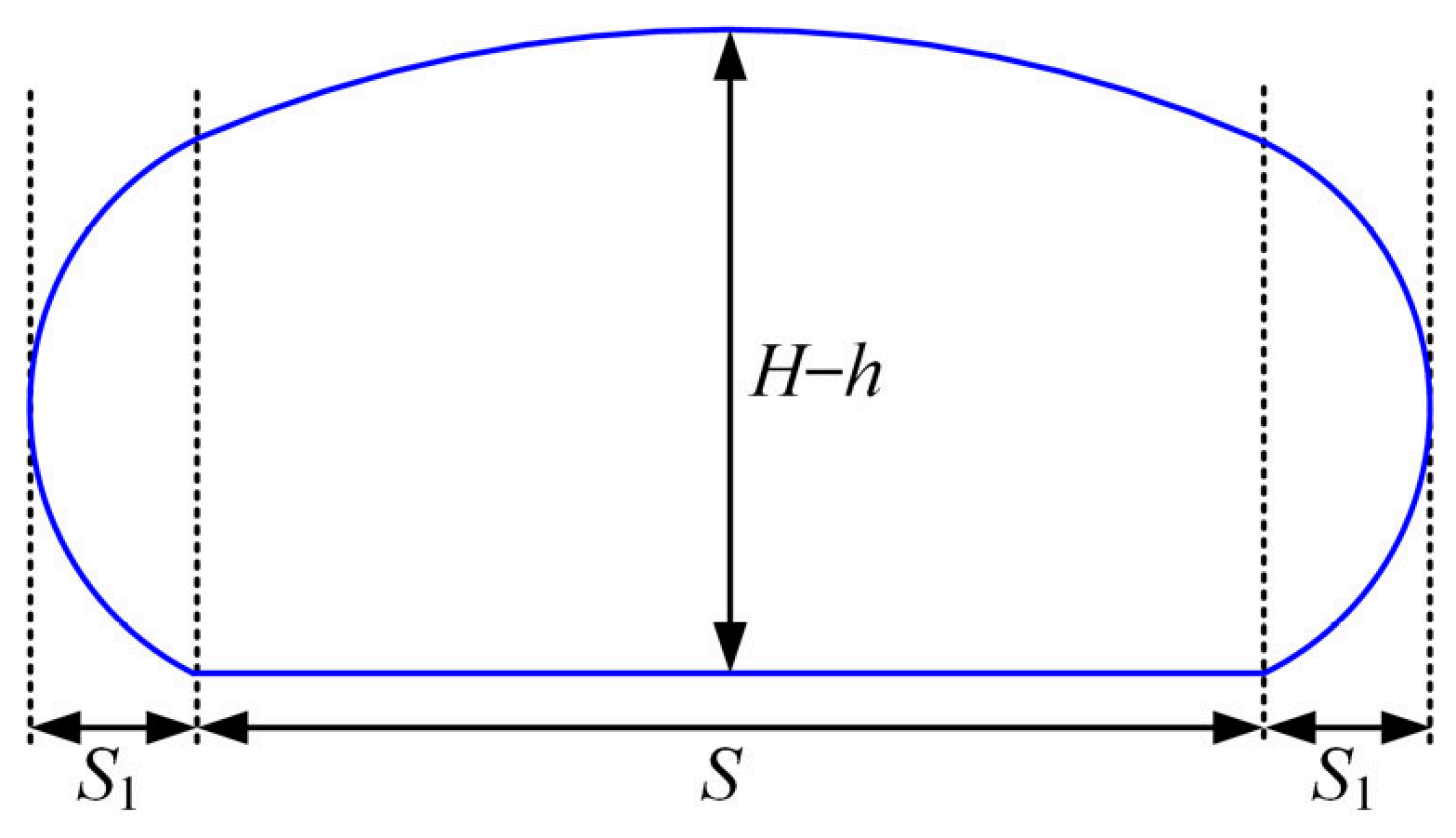
The principle of quintic polynomial bionic trajectory.

The quintic polynomial bionic trajectory is shown in Figure 17; *S* denotes the step length for forward movement, and *S*_1_ denotes the step length for retraction. Due to the presence of a retraction trajectory, the force exerted on the robot upon ground contact is oriented nearly parallel to the ground surface, thereby effectively mitigating vertical impact forces.

**Figure 18:**
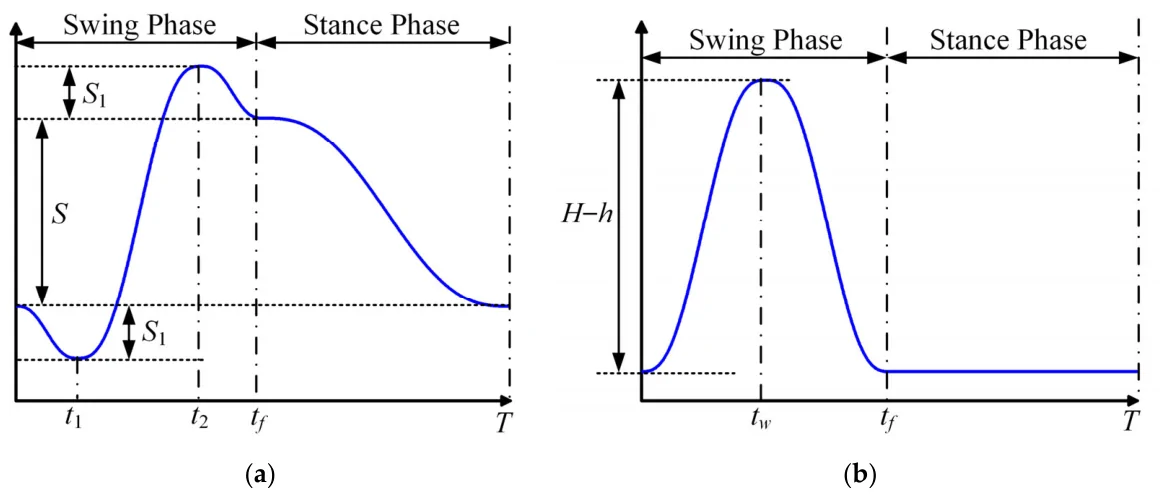
The quintic polynomial bionic trajectory along the *x*-axis and *y*-axis. (**a**) Along the *x*-axis. (**b**) Along the *y*-axis.

Within one cycle, the quintic polynomial bionic trajectory exhibits displacements along the *x*-axis and *y*-axis, as shown in Figure 18a and b, respectively. The robot’s foot lifts off the ground at time *t* = 0 and makes contact again at time *t* = *t_f_*. The interval [0, *t_f_*] constitutes the swing phase, while [*t_f_*, *T*] corresponds to the stance phase. Within the swing phase, [0, *t*_1_] denotes the trajectory retraction segment, [*t*_1_, *t*_2_] denotes the stepping segment, and [*t*_2_, *t_f_*] denotes the trajectory retraction segment. The foot reaches its maximum height at time *t* = *t_w_*, subsequently descends under gravity, and finally contacts the ground at time *t* = *t_f_*.

The *x*-direction expression of the quintic polynomial bionic trajectory is

(45) $$x=\left\{\begin{array}{l} -10S_{1}{\left(\frac{t}{t_{1}}\right)}^{3}+15S_{1}{\left(\frac{t}{t_{1}}\right)}^{4}-6S_{1}{\left(\frac{t}{t_{1}}\right)}^{5},\;\;\;\;\;\;\;\;\;\;\;\;\;\;\;\;\;\;\;\;\;\;\;\;\;\;\;\;\;\;\;\;\;\;\;\;\;\;\;\;\;\;\;\;\;\;\;\;\;\;\;\;\;\;\;\;\;\;\;\;\;\;\;\;\;\;\;\;\;\;\;\;\;\;(0\leq t\leq t_{1})\\ -S_{1}+10(2S_{1}+S){\left(\frac{t-t_{1}}{t_{2}-t_{1}}\right)}^{3}-15(2S_{1}+S){\left(\frac{t-t_{1}}{t_{2}-t_{1}}\right)}^{4}+6(2S_{1}+S){\left(\frac{t-t_{1}}{t_{2}-t_{1}}\right)}^{5},\;\;\;(t_{1}\leq t\leq t_{2})\\ S_{1}+S-10S_{1}{\left(\frac{t-t_{2}}{t_{f}-t_{2}}\right)}^{3}+15S_{1}{\left(\frac{t-t_{2}}{t_{f}-t_{2}}\right)}^{4}-6S_{1}{\left(\frac{t-t_{2}}{t_{f}-t_{2}}\right)}^{5},\;\;\;\;\;\;\;\;\;\;\;\;\;\;\;\;\;\;\;\;\;\;\;\;\;\;\;\;\;\;\;\;\;\;\;\;\;(t_{2}\leq t\leq t_{f})\\ S-10S{\left(\frac{t-t_{f}}{T-t_{f}}\right)}^{3}+15S{\left(\frac{t-t_{f}}{T-t_{f}}\right)}^{4}-6S{\left(\frac{t-t_{f}}{T-t_{f}}\right)}^{5}.\;\;\;\;\;\;\;\;\;\;\;\;\;\;\;\;\;\;\;\;\;\;\;\;\;\;\;\;\;\;\;\;\;\;\;\;\;\;\;\;\;\;\;\;\;\;\;\;\;\;\;\;\;(t_{f}\leq t\leq T) \end{array}\right.$$

The *y*-direction expression of the quintic polynomial bionic trajectory is

(46) $$y=\left\{\begin{array}{l} H+10(h-H){\left(\frac{t}{t_{w}}\right)}^{3}-15(h-H){\left(\frac{t}{t_{w}}\right)}^{4}+6(h-H){\left(\frac{t}{t_{w}}\right)}^{5},\;\;\;\;\;\;\;\;\;\;\;\;\;\;\;\;\;\;\;\;\;\;\;\;\;\;(0\leq t\leq t_{w})\\ h+10(H-h){\left(\frac{t-t_{w}}{t_{f}-t_{w}}\right)}^{3}-15(H-h){\left(\frac{t-t_{w}}{t_{f}-t_{w}}\right)}^{4}+6(H-h){\left(\frac{t-t_{w}}{t_{f}-t_{w}}\right)}^{5},\;\;\;\;(t_{w}\leq t\leq t_{f})\\ H.\;\;\;\;\;\;\;\;\;\;\;\;\;\;\;\;\;\;\;\;\;\;\;\;\;\;\;\;\;\;\;\;\;\;\;\;\;\;\;\;\;\;\;\;\;\;\;\;\;\;\;\;\;\;\;\;\;\;\;\;\;\;\;\;\;\;\;\;\;\;\;\;\;\;\;\;\;\;\;\;\;\;\;\;\;\;\;\;\;\;\;\;\;\;\;\;\;\;\;\;\;\;\;\;\;\;\;\;\;\;\;\;\;\;\;\;\;\;\;\;\;\;(t_{f}\leq t\leq T) \end{array}\right.$$

**Figure 19:**
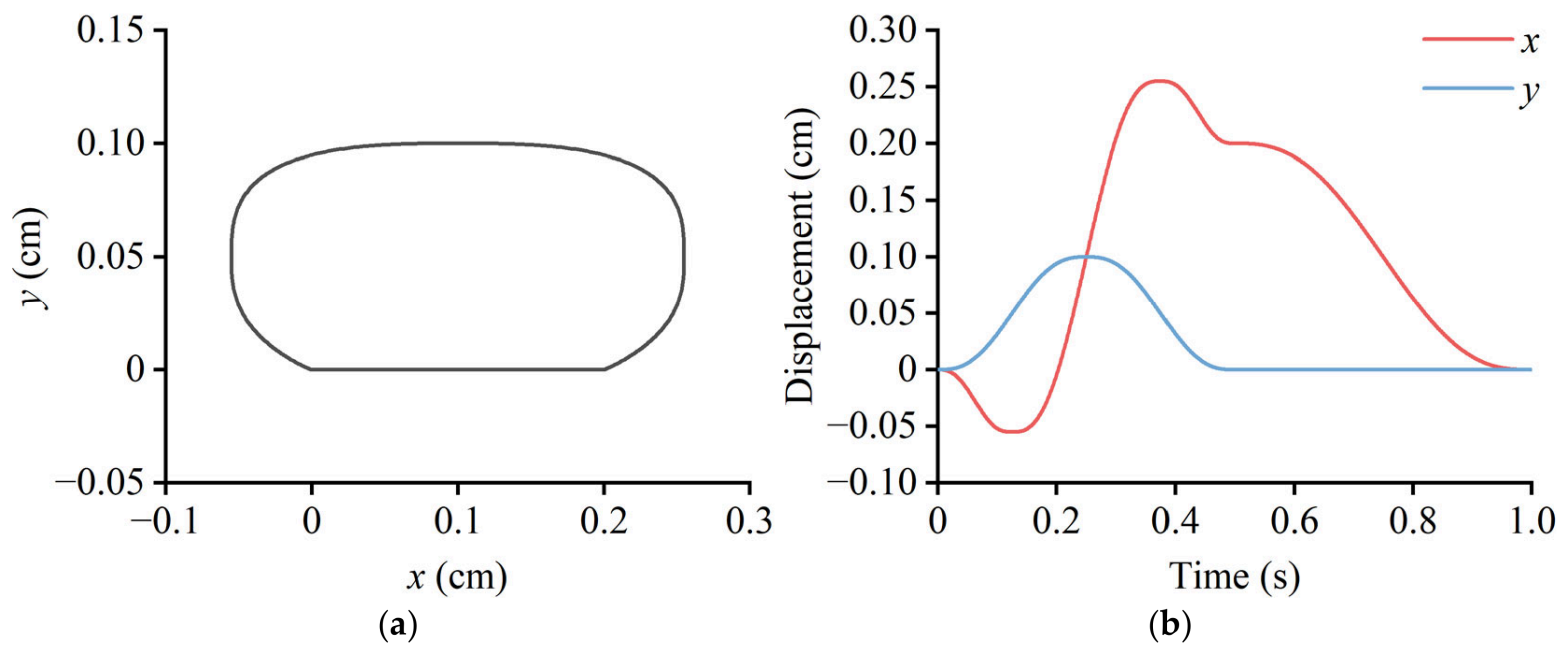
The desired quintic polynomial bionic trajectory and its projection along the *x*-axis and *y*-axis. (**a**) The desired quintic polynomial bionic trajectory. (**b**) The projection of the foot trajectory.

The robot’s leg-lifting height is 1 mm, the step length is 2 mm, and the retraction step length *S*_1_ is 0.55 mm. The corresponding time intervals are defined as *t*_1_ = T/8, *t*_2_ = 3T/8, *t_f_* = T/2, and *t_w_* = T/4. Based on the kinematic model [19], the quintic polynomial bionic trajectory is illustrated in Figure 19a, while its displacement variations along the *x*-axis and *y*-axis are presented in Figure 19b.

#### 2.3.2. Locomotion Control Strategy Design

**Figure 20:**
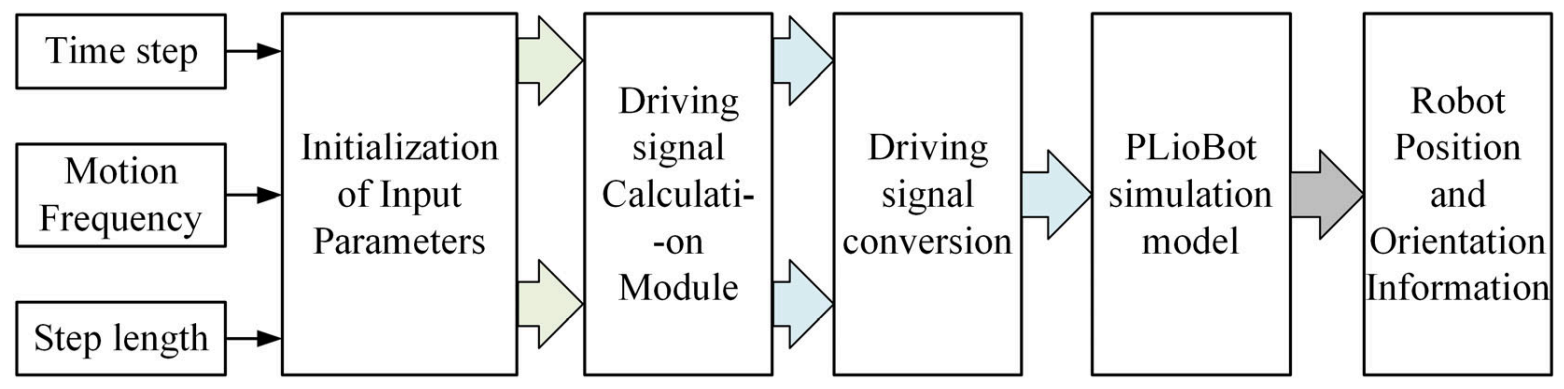
The principle of the locomotion simulation model.

The principle of the robot locomotion simulation model is shown in Figure 20. The driving displacement rate signals serve as the driving inputs of the PLioBot simulation model, while the robot’s position and orientation constitute its outputs. The time step is fixed at 0.001 s, and both the step length and locomotion frequency are specified by the controller. The driving signals corresponding to each time step at the robot’s feet are generated by the driving signal calculation module. The driving signals undergo amplitude limiting and signal conversion before being fed into the PLioBot model, which then generates the robot’s simulated position and orientation.

**Figure 21:**
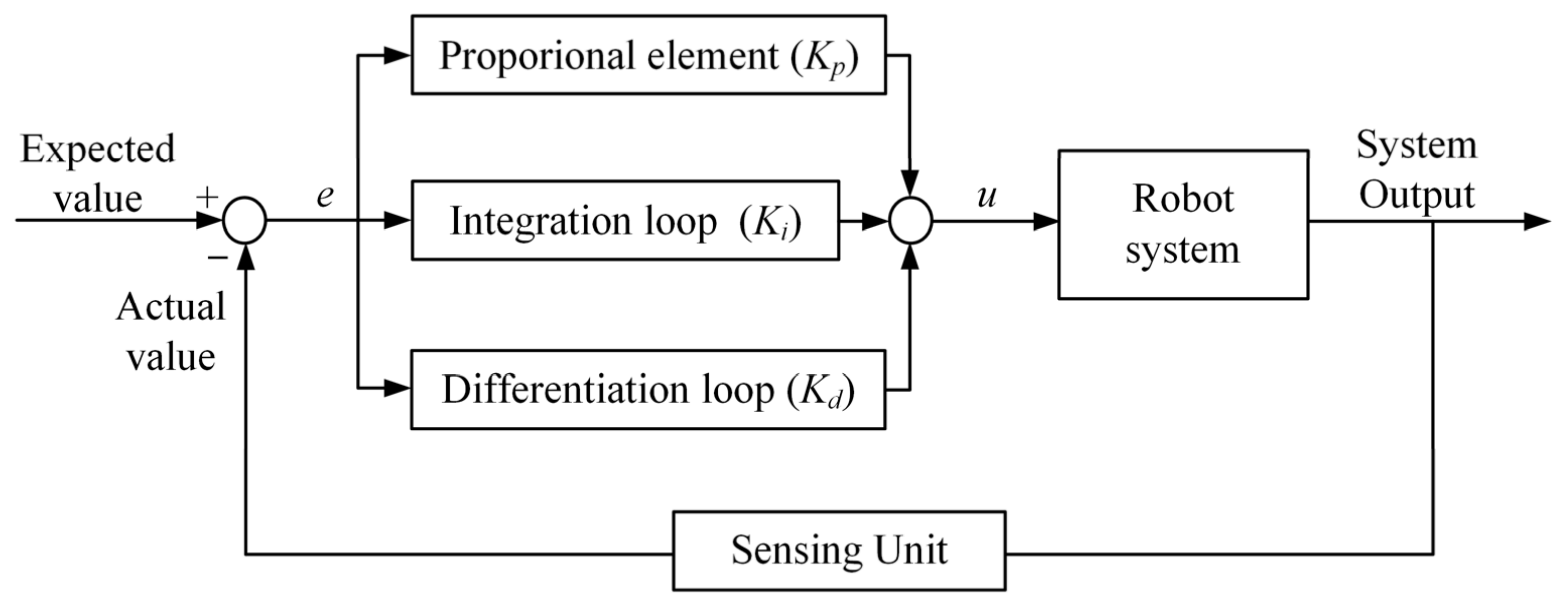
The principle of the PID closed-loop controller.

The proportional–integral–derivative (PID) algorithm is a widely adopted control algorithm and demonstrates strong applicability potential in insect-scale micro crawling robots due to its structural simplicity, ease of implementation, and independence from an accurate robot model [9,37,38,39]. The principle of the PID control system is illustrated in Figure 21.

The PID closed-loop control system primarily consists of three components: proportional element, integration loop, and differentiation loop. The ideal continuous-time PID control law is mathematically expressed as

(47) $$u(t)=K_{p}e(t)+K_{i}\int_{0}^{t}e(\tau )d\tau +K_{d}\frac{de(t)}{dt},$$

where *e* denotes the error between the system output and the expected value. *K_p_*, *K_i_*, *K_d_* represent the proportional, integral, and derivative gains of the PID controller.

**Figure 22:**
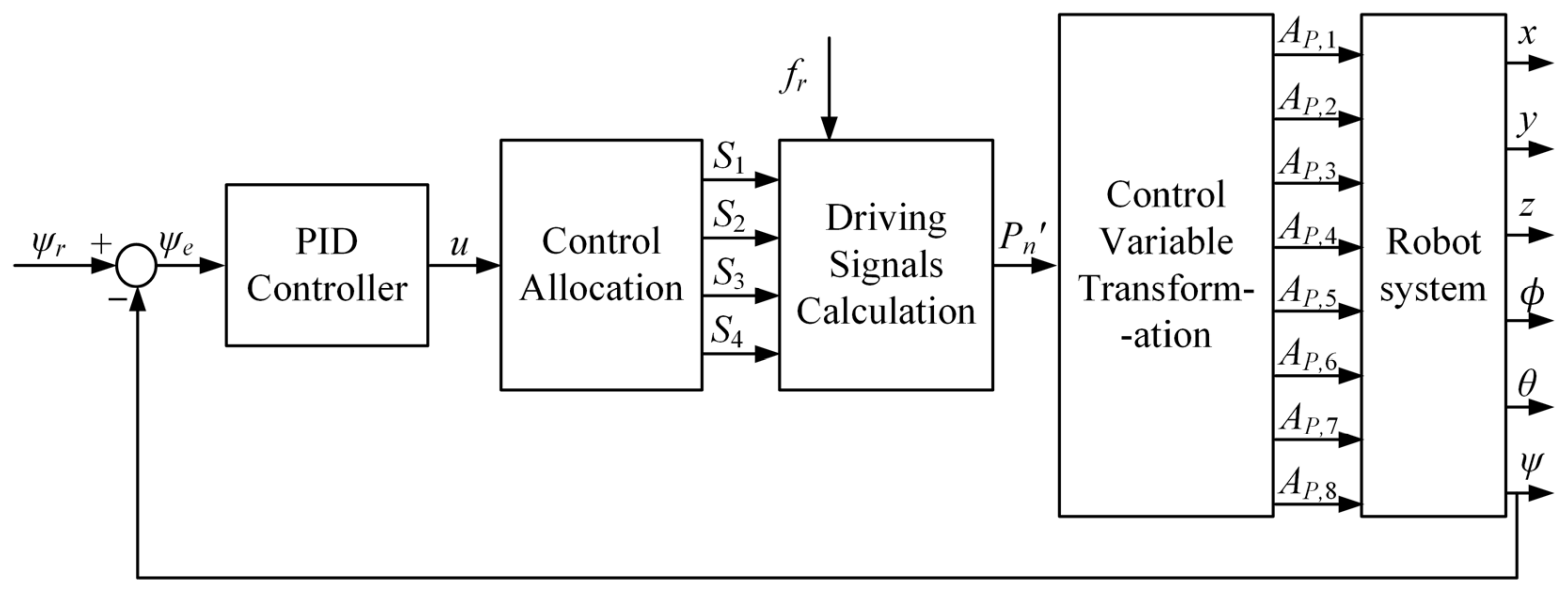
The principle of the PLioBot’s closed-loop controller.

**Figure 23:**
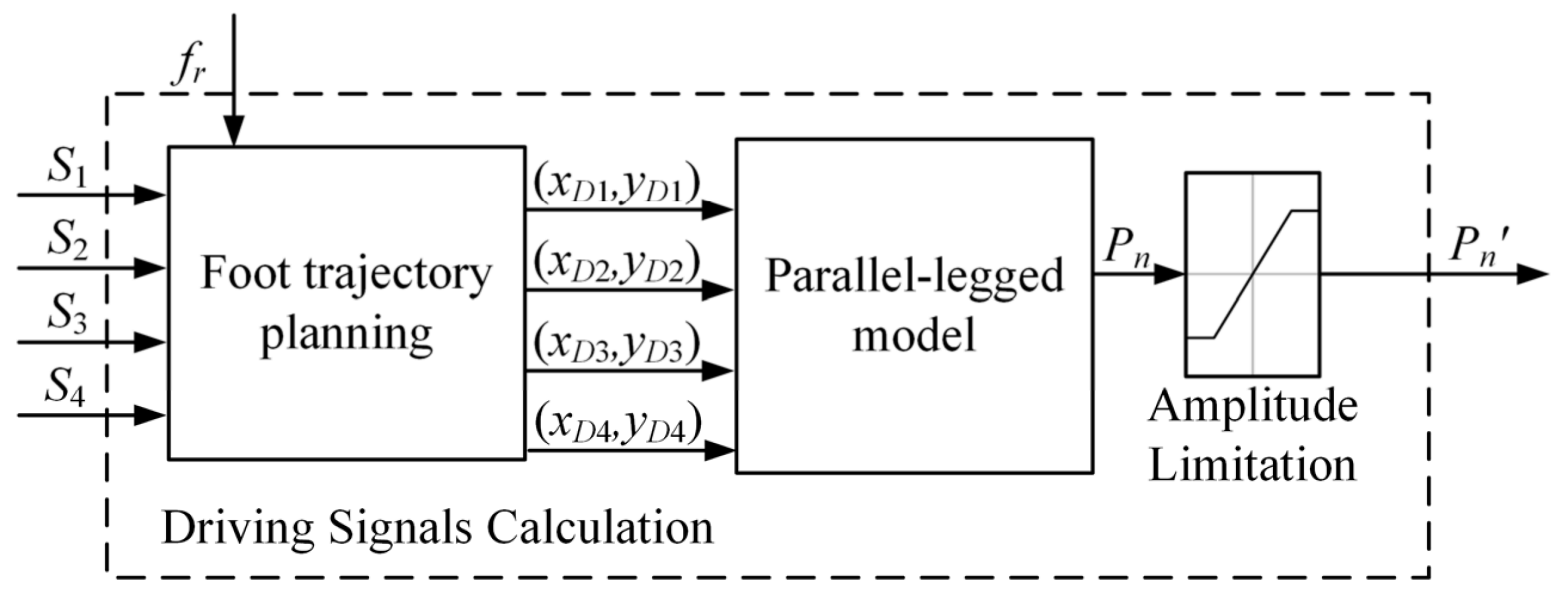
The driving signals calculation module of the closed-loop controller.

The robot closed-loop controller is designed based on the PID control algorithm, and its control principle is illustrated in Figure 22. The driving signals calculation module is as shown in Figure 23. For a given desired yaw angle *ψ*, the yaw angle error *ψ_e_* is computed as the difference between *ψ* and the measured yaw angle *ψ* obtained from robot simulation feedback. This error signal *ψ_e_* is fed into the PID controller, which generates the corresponding control input for robot actuation, thereby establishing the closed-loop PID control system. The PID controller generates the control signal *u* to regulate the step amplitudes *S*_1_, *S*_2_, *S*_3_, and *S*_4_ of the PLioBot’s legs. Subsequently, foot trajectory planning is performed to compute the foot positions (*x_D_*, *y_D_*) in the leg-fixed coordinate frame. Based on the inverse kinematics theory of the robot’s leg [19], the displacement *P_n_*′ of the piezoelectric actuator is computed. Subsequently, with the control variable transformation, the driving signals are obtained through amplitude limiting and signal transformation. These driving signals are then fed into the robot system to achieve regulation of the robot’s motion posture angle.

In the control allocation module, the PID control signal *u* serves as the input, while the four-leg step amplitudes *S*_1_, *S*_2_, *S*_3_, and *S*_4_ constitute the outputs. The relationship of the control allocation is expressed as

(48) $$\left\{\begin{array}{l} S_{1}=S_{2}=S_{0}+u,\\ S_{3}=S_{4}=S_{0}-u,\\ \left|S_{1},S_{2},S_{3},S_{4}\right|<S_{\max}, \end{array}\right.$$

where *S*_0_ is the base step length and *S*_max_ denotes the maximum step length achievable by the robot’s leg. For the PLioBot, *S*_max_ is set to 0.2 cm.

## 3. Results

### 3.1. Robot Simulation Model and Parameters

**Figure 24:**
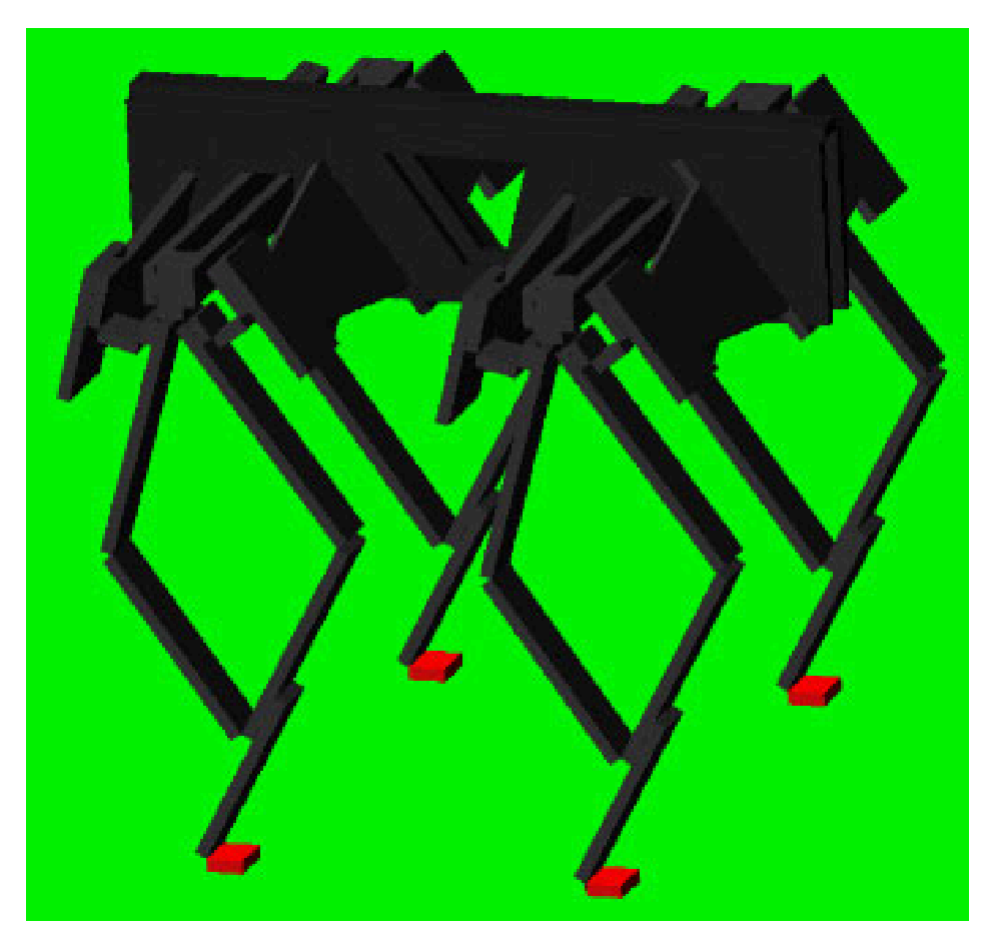
The locomotion simulation model of the PLioBot.

The locomotion simulation model of the PLioBot is as illustrated in Figure 24. The flexible hinges are modeled as revolute joints with torsional stiffness. To facilitate the validation of the control strategy, the piezoelectric actuators are modeled as prismatic joints. The driving inputs to the robot locomotion simulation model are the displacement rates of the eight piezoelectric actuators. Based on the PRBM of the flexible hinges, the equivalent stiffness value of the torsional spring at the parallel-legged joints is assigned, as listed in Table 3. In the simulation model, the densities of the robot’s components are determined by computing the ratio of their actual masses to their respective volumes. The specific values are provided in Table 4.

The robot generates contact forces upon interaction with the ground, and quadrupedal locomotion involves both frictional interaction and impact collisions with the ground. The contact parameters for the PLioBot feet to the ground in the simulation model are listed in Table 5.

### 3.2. Open-Loop Simulation of the Foot Trajectory

**Figure 25:**
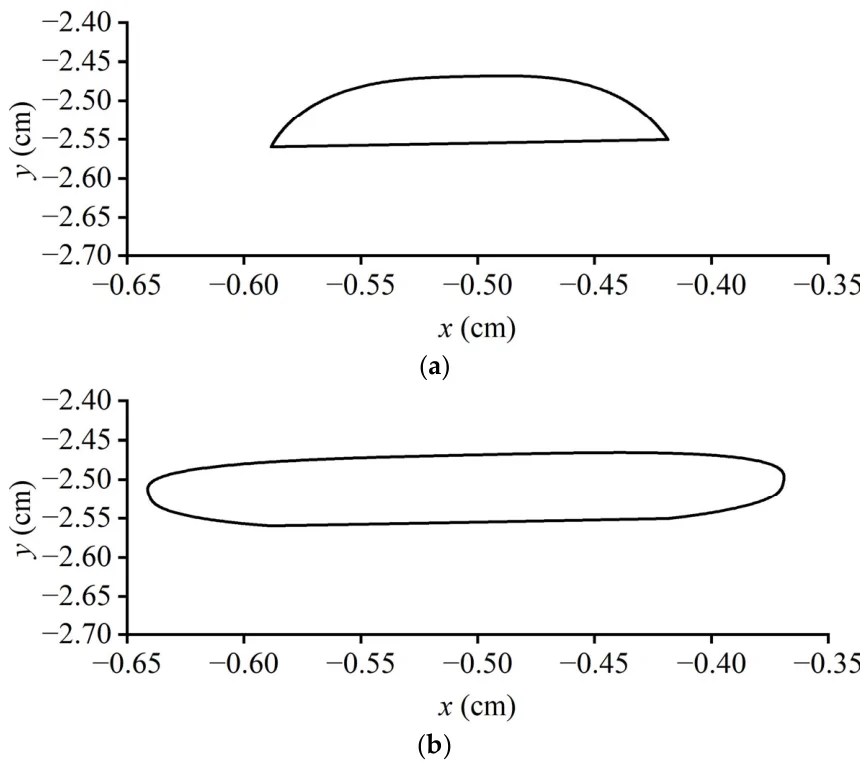
The resulting foot trajectories obtained from the simulation model. (**a**) The compound cycloidal trajectory of the PLioBot simulation model. (**b**) The quintic polynomial bionic trajectory of the PLioBot simulation model.

The compound cycloidal trajectory and the quintic polynomial bionic trajectory are implemented as the foot motion inputs, respectively. The resulting foot trajectories obtained from the simulation model are presented in Figure 25a and b, respectively.

**Figure 26:**
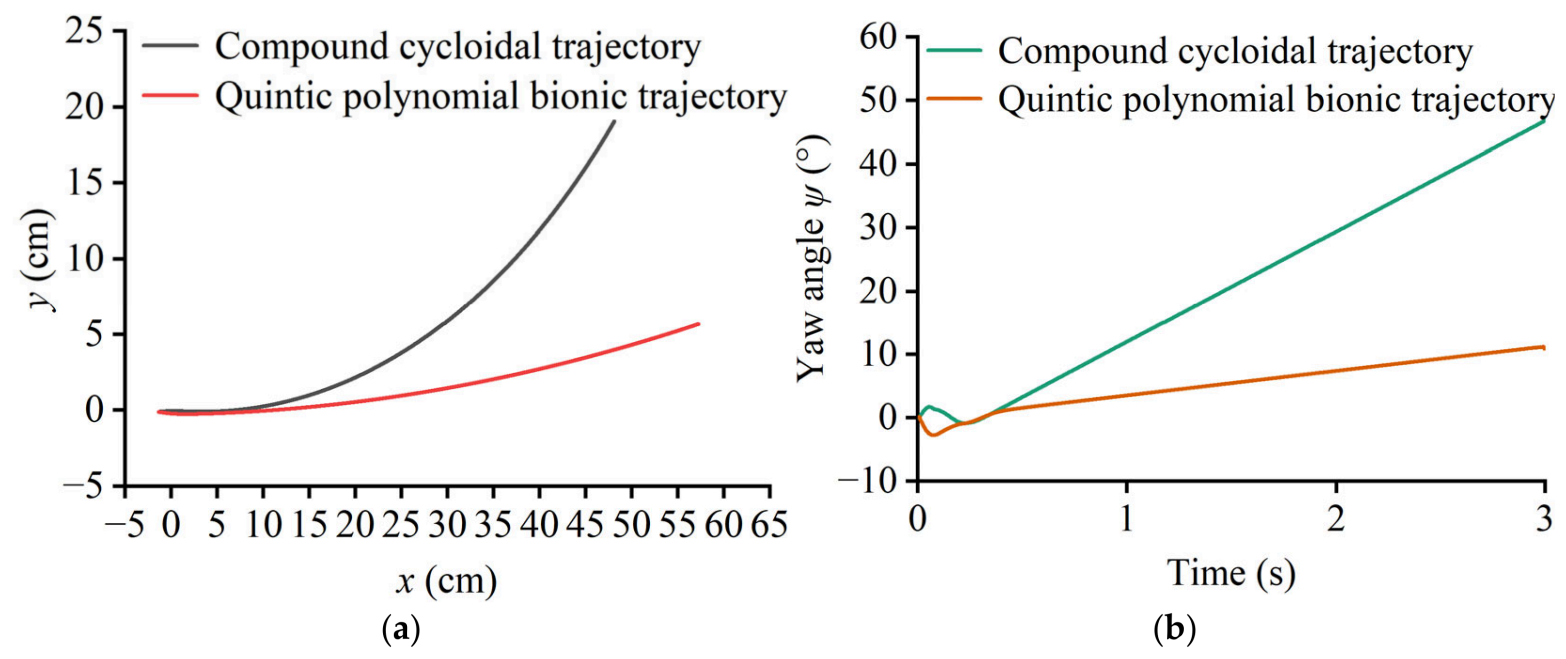
The robot’s position and yaw angle evolution with the different foot trajectories in the pace gait. (**a**) The robot’s positional trajectory. (**b**) The robot’s yaw angle evolution.

PLioBot is configured to operate in the pace gait at 40 Hz. The corresponding positional trajectory and yaw angle evolution under the two foot trajectory inputs are shown in Figure 26a and b, respectively. During open-loop locomotion with the pace gait, the maximum yaw angle induced by the quintic polynomial bionic trajectory is smaller than that generated by the composite cycloidal trajectory.

**Figure 27:**
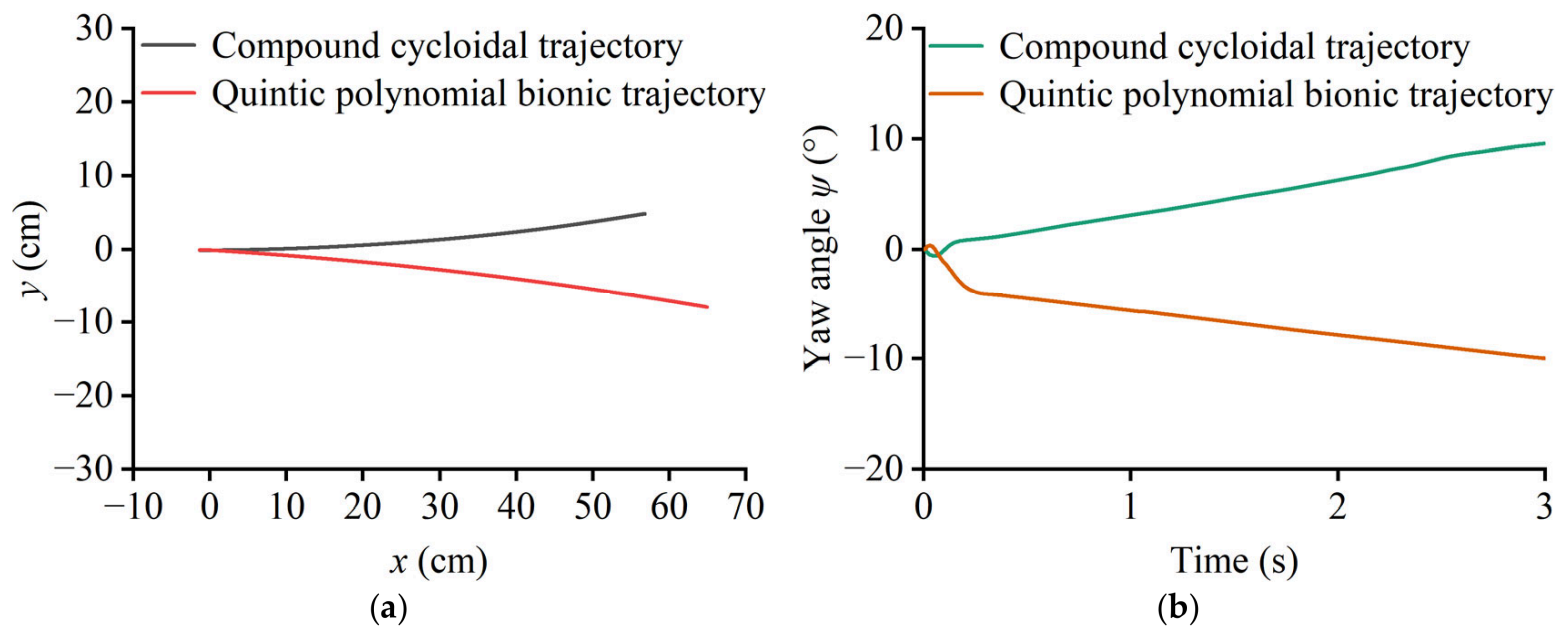
The robot’s position and yaw angle evolution with the different foot trajectories in trot gait. (**a**) The robot’s positional trajectory. (**b**) The robot’s yaw angle evolution.

Under identical trajectory and frequency inputs, the PLioBot is configured to execute the trot gait. Its positional trajectory and yaw angle variation are shown in Figure 27a and b, respectively. For both foot trajectories, the yaw angle deviation during trot gait remains within ±10°.

Since the robot’s closed-loop control strategy primarily relies on differential control of left- and right-side step lengths, it is not directly linked to the robot’s foot trajectory. Therefore, compared with the composite cycloidal trajectory, the quintic polynomial bionic trajectory yields superior straight-line locomotion performance under the pace gait. The quintic polynomial bionic trajectory is thus selected as the foot trajectory input for PLioBot’s locomotion control simulation.

**Figure 28:**
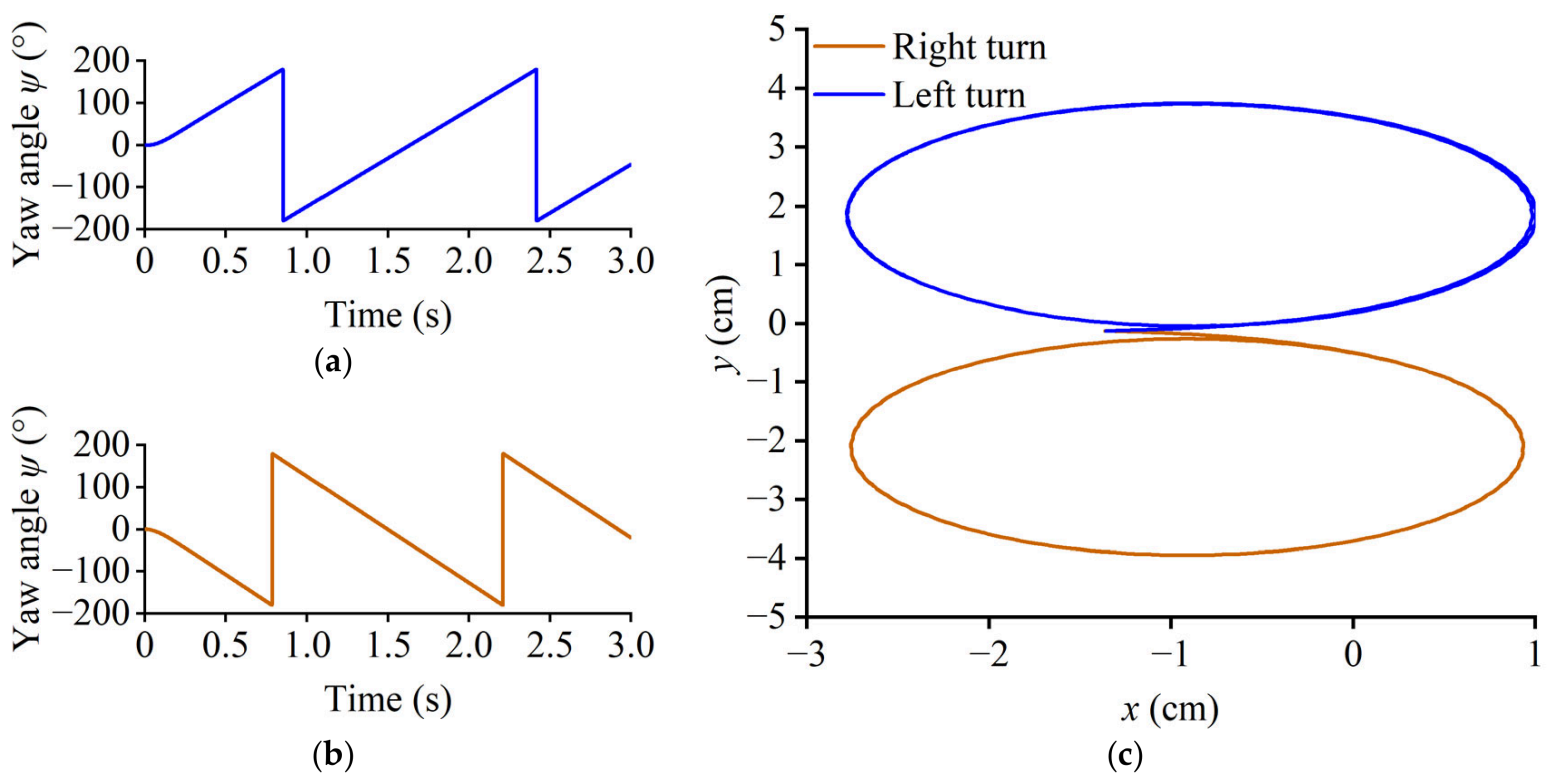
The left-turn and right-turn motions of the PLioBot. (**a**) The yaw angle variation in the robot during the left-turn motion. (**b**) The yaw angle variation in the robot during the right-turn motion. (**c**) The position coordinates variation in the robot during the steering motion.

The robot employs the pace gait with a locomotion frequency of 40 Hz. When the step length of the left leg is set to 0.02 cm and that of the right leg to 0.2 cm, the robot executes a left-turn motion. The robot’s yaw angle variation is shown in Figure 28a. Conversely, when the left-leg step length is set to 0.2 cm and the right-leg step length to 0.02 cm, the robot performs a right-turn motion. The associated yaw angle variation is presented in Figure 28b. The positional trajectories of the PLioBot during left and right turns are illustrated in Figure 28c.

**Figure 29:**
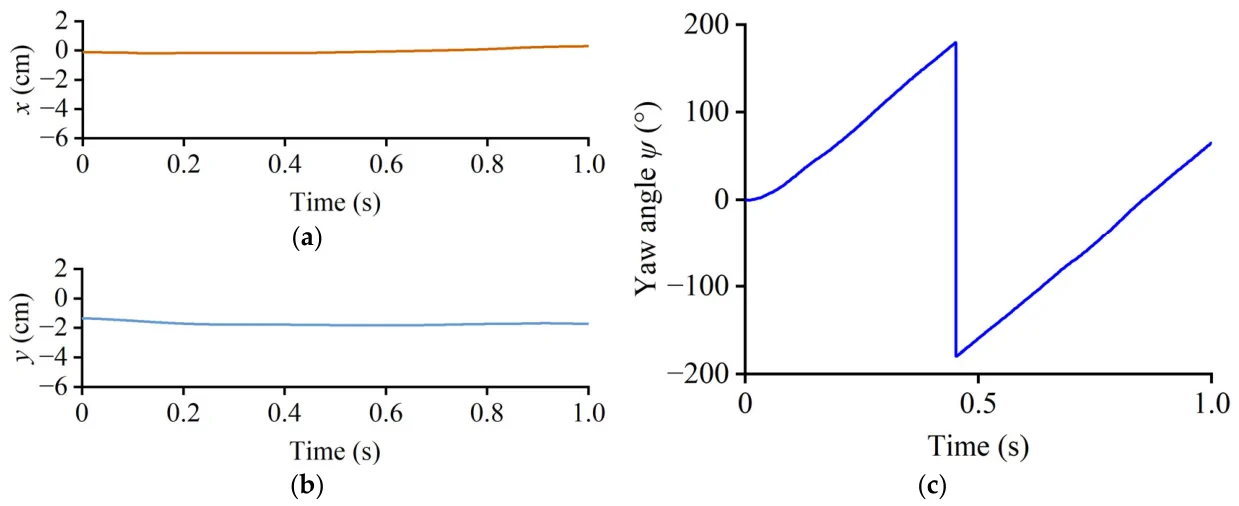
The in-place turning motion of the PLioBot. (**a**) The *x*-coordinate of the PLioBot. (**b**) The *y*-coordinate of the PLioBot. (**c**) The yaw angle variation in the robot.

**Figure 30:**
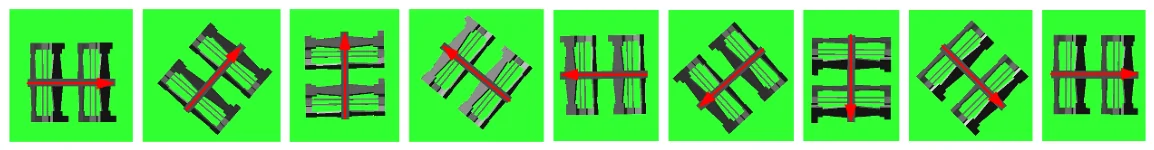
The simulation results of the robot’s in-place turning locomotion. The red arrow indicates the robot’s forward direction.

The motion frequency of the robot is set to 40 Hz, with a step length of 0.2 cm for the left leg and −0.2 cm for the right leg. The negative sign denotes the direction of stepping. Under this configuration, the robot executes in-place turning. The position coordinates variation of *x* and *y* are illustrated in Figure 29a and b, respectively. The corresponding yaw angle variation in the robot is shown in Figure 29c. The simulation results of the robot’s motion are presented in Figure 30.

### 3.3. Closed-Loop Simulation of the Control Strategy

**Figure 31:**
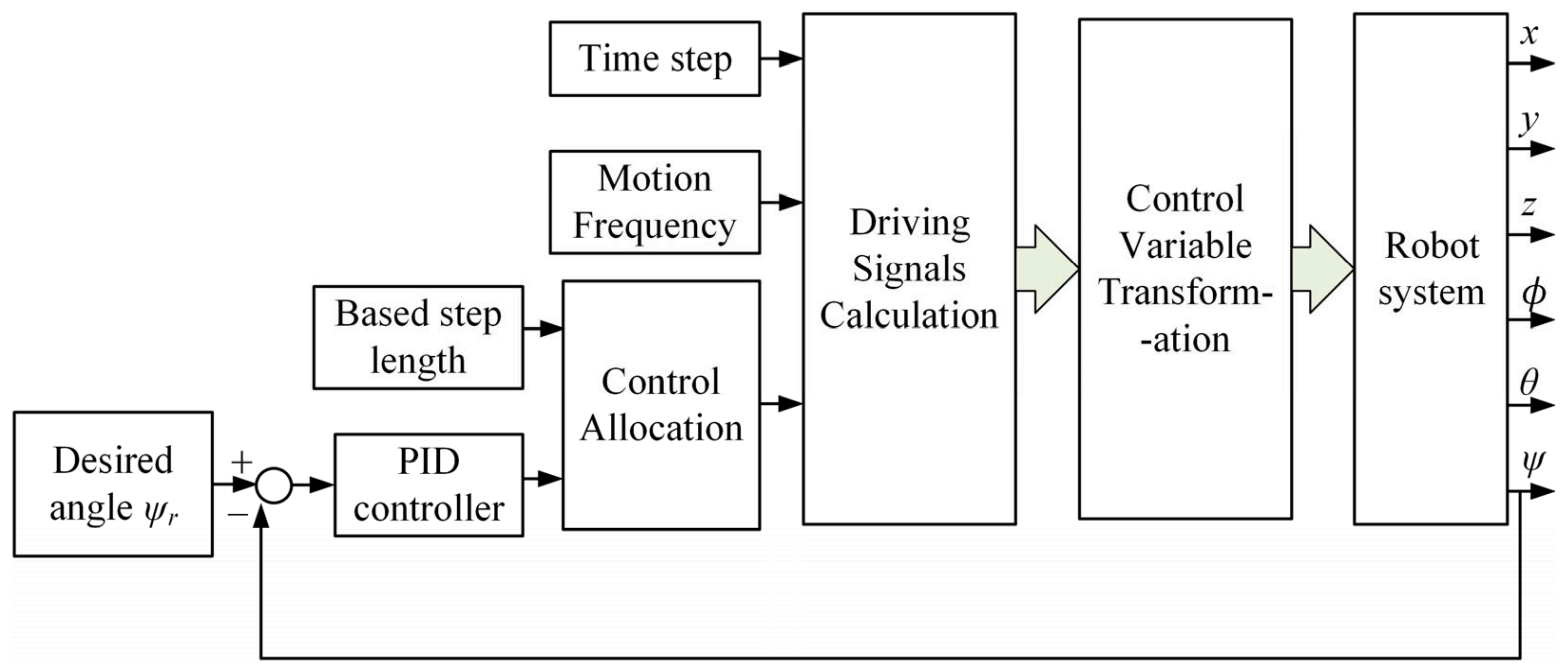
The principle of the robot locomotion co-simulation system.

**Figure 32:**
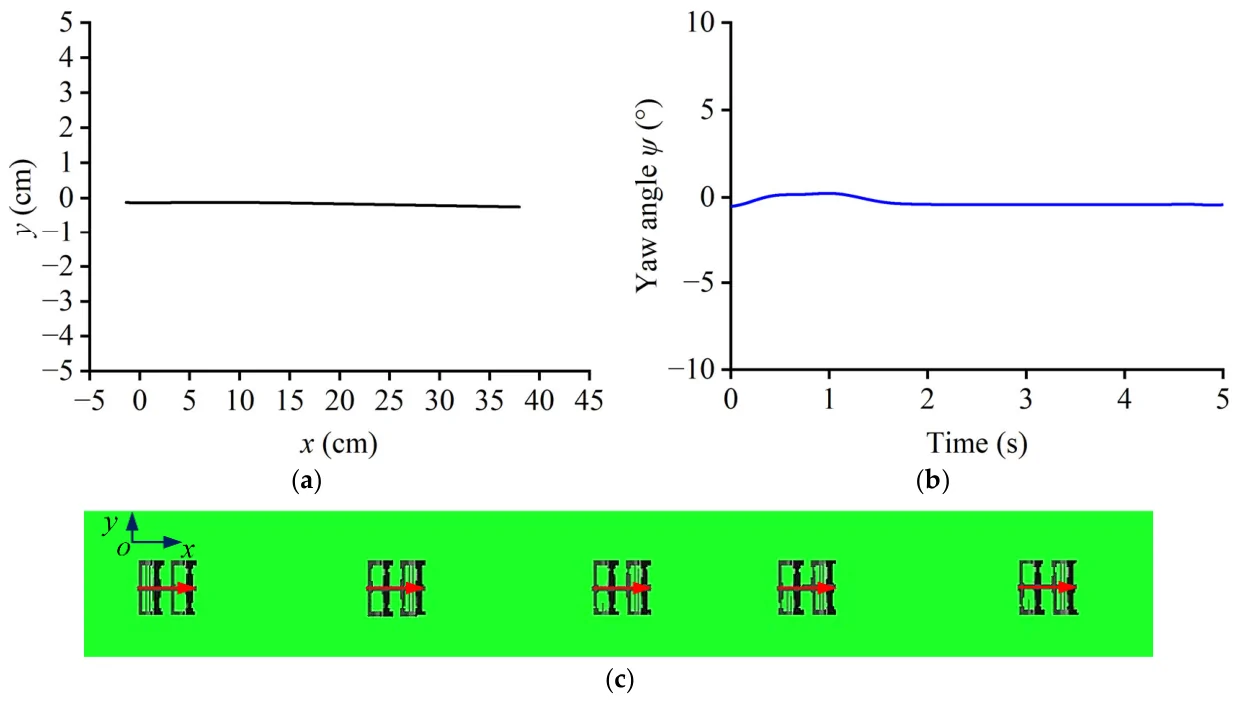
The simulation results of the straight-line locomotion control. (**a**) The positional coordinate variation in the PLioBot. (**b**) The yaw angle variation in the PLioBot. (**c**) The simulation results of the robot’s straight-line motion. The red arrow indicates the robot’s forward direction.

To validate the proposed control strategy, the locomotion co-simulation system of the PLioBot is developed, and its principle is as illustrated in Figure 31. The yaw angle is fixed at 0°, and the robot is commanded to execute straight-line locomotion. The base step length of the robot is set to 0.1 cm, and the actuation frequency is configured at 40 Hz. The discrete-time PID controller is implemented with the gains *K_p_* = 1.5 × 10^−4^, *K_i_* = 8 × 10^−6^, and *K_d_* = 1 × 10^−10^, using a sampling time of *T_s_* = 0.001 s. Under these conditions, the simulation results of the straight-line locomotion control are presented in Figure 32a–c. The robot is capable of executing straight-line motion, with the maximum lateral displacement deviation being 0.4 cm.

**Figure 33:**
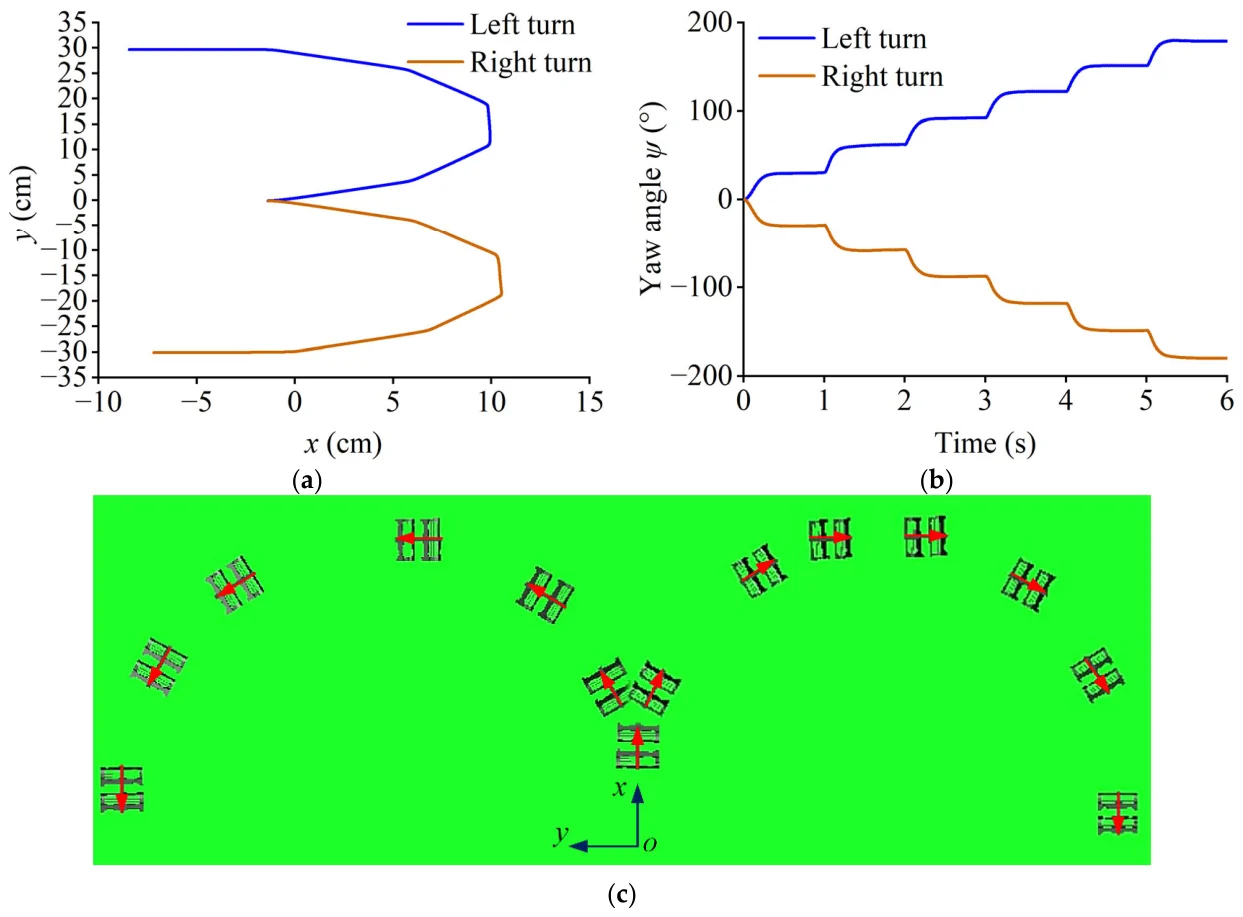
The multi-angle steering performance of the PLioBot. (**a**) The positional coordinate variation in the PLioBot. (**b**) The yaw angle variation in the PLioBot. (**c**) The simulation results of the robot’s multi-angle steering motion. The red arrow denotes the robot’s forward direction.

Maintain a constant motion frequency of 40 Hz and a base step length of 0.1 cm. The robot’s steering angle is designed to increment in steps of 30°, ranging from 0° to 180°. The multi-angle steering performance of the PLioBot is systematically evaluated under these conditions. The corresponding variations in the robot’s position during multi-angle steering is presented in Figure 33a. The yaw angle evolution and the simulation results are presented in Figure 33b and c, respectively. The PID controller is implemented with the gains *K_p_* = 4 × 10^−5^, *K_i_* = 5 × 10^−7^, and *K_d_* = 1 × 10^−9^. For multiple angular adjustments, the robot achieves the target angle within 0.2 s and maintains stable motion at the specified angle.

The robot’s steering angles are designed with increments of 90°, spanning from 0° to 360°. The robot’s motion control frequency and base step length are maintained at 40 Hz and 0.1 cm, respectively. The PID parameters are identical to those used in the straight-line locomotion control. *ψ* is the yaw angle with the angular range of [−180°, 180°]. To prevent abrupt jumps in the yaw angle error *ψ_e_*, the yaw angle error *ψ_e_* is defined as

(49) $$\;\psi_{e}=\mathrm{mod}(\psi_{r}-\psi +180,\;360)-180,$$

where the mod denotes the modulo operation.

**Figure 34:**
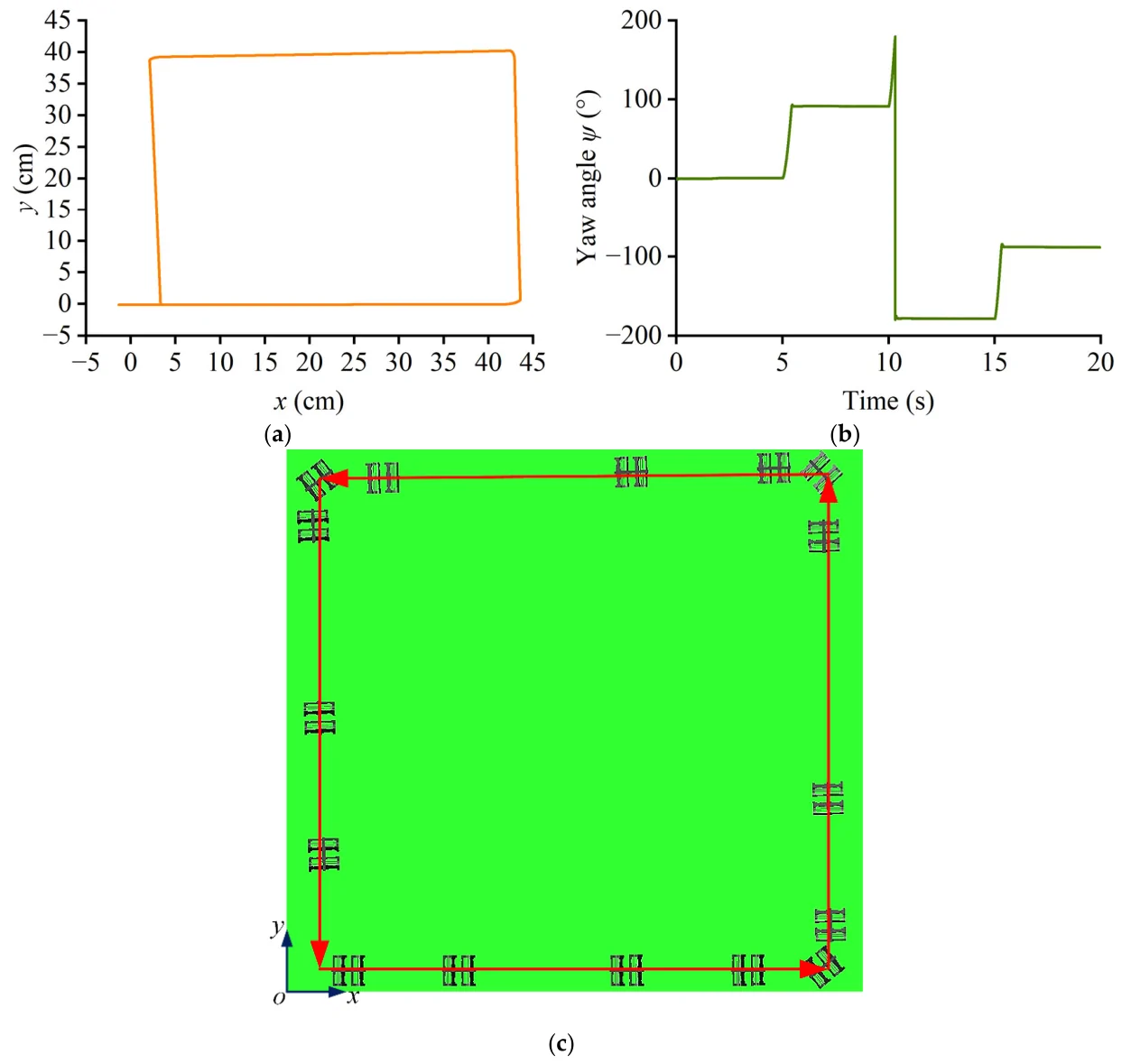
The robot motion with a quadrilateral trajectory. (**a**) The positional coordinate variation in the PLioBot. (**b**) The yaw angle variation in the PLioBot. (**c**) The simulation results of the robot. The red arrow indicates the robot’s forward direction.

Under this configuration, the robot generates a quadrilateral trajectory. Its positional trajectory variation, yaw angle, and simulation motion results are illustrated in Figure 34a, b, and c, respectively. The maximum steady-state yaw angle error over the robot’s three 90° turns is 1.5°. The robot exhibits a positional deviation of 4.21 cm along the *x*-axis and 0.06 cm along the *y*-axis relative to its initial position.

**Figure 35:**
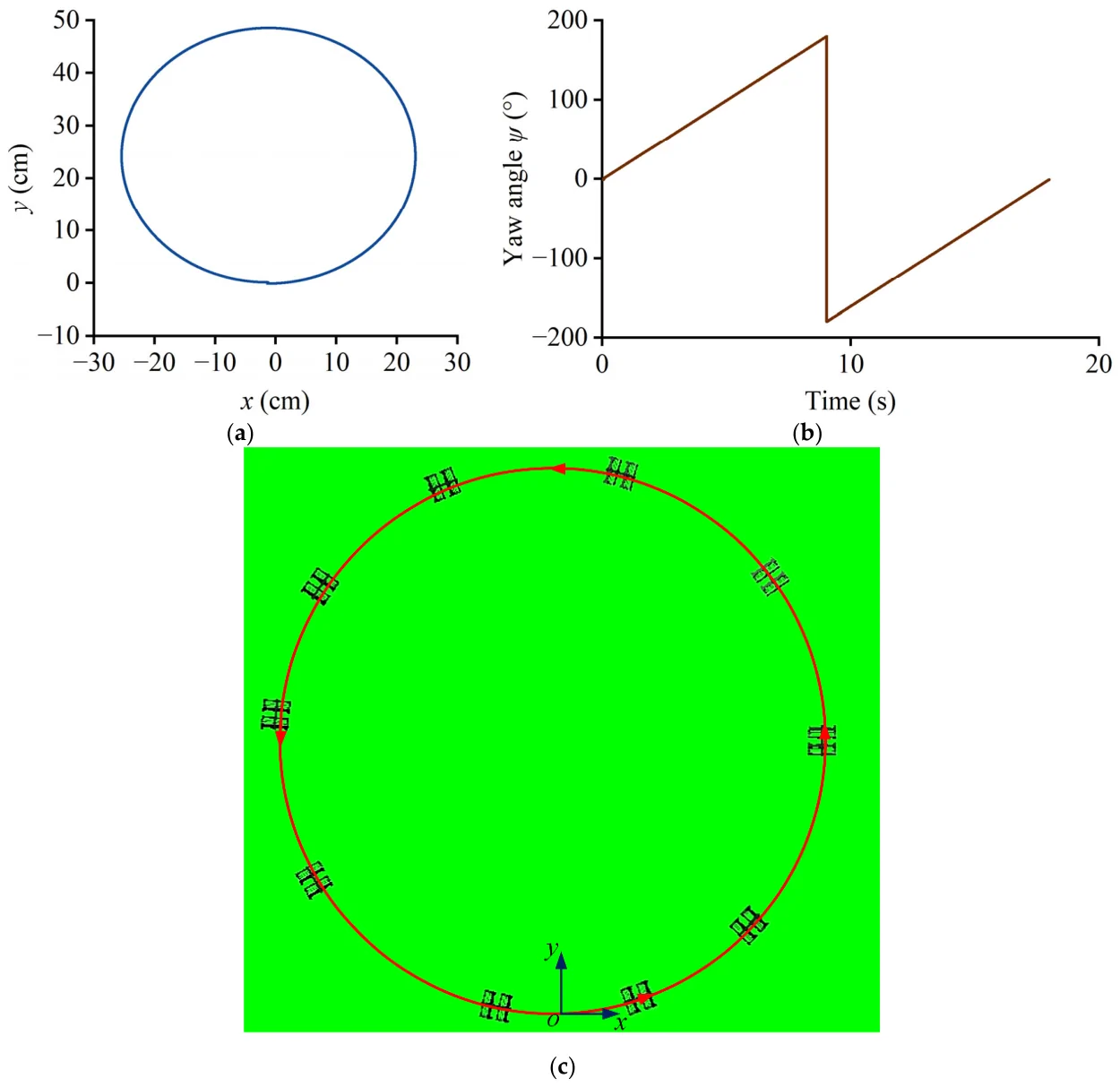
The robot motion with a circular trajectory. (**a**) The positional coordinate variation in the PLioBot. (**b**) The yaw angle variation in the PLioBot. (**c**) The simulation motion behavior. The red arrow indicates the robot’s forward direction.

The angular velocity is set to 20°/s, and the robot executed a circular trajectory. The actuation frequency is configured at 40 Hz, with a base step length of 0.1 cm. The PID controller parameters and sampling time are identical to those employed in the straight-line locomotion control. The positional coordinate, yaw angle variation, and the simulation motion behavior for the circular locomotion control of the PLioBot are presented in Figure 35a–c. The robot is capable of returning to its initial position, thereby generating a closed circular trajectory. The robot’s end position has a deviation of 0.2 cm in the *x*-axis and 0.2 cm in the *y*-axis relative to the start position.

## 4. Discussion

Controlled locomotion of insect-scale microrobots is fundamentally constrained by limitations in robot modeling accuracy and onboard computational capacity. Composed of multiple composite raw materials and a rigid–flexible coupled structure, and fabricated via robot-specific manufacturing processes, the microrobotic system exhibits strongly nonlinear behavior, making it challenging to establish an accurate mathematical model. The contradiction between low-computational microprocessing systems and complex control algorithms forces the microrobot to rely on large external computer systems, precluding the wireless operation.

Previous work has introduced a novel insect-scale micro crawling robot (PLioBot). However, the transition from open-loop actuation to closed-loop feedback control remains a critical bottleneck for achieving untethered operation of PLioBot [19]. By analyzing the transmission processes during robotic locomotion, this study establishes the mathematical theoretical model of PLioBot’s motion. This study analyzes the piezoelectric actuator model, the flexible hinge model, the parallel-legged mechanism dynamic model, the foot–ground contact model, and the differential-stride turning locomotion model to establish a mathematical theoretical model of the PLioBot locomotion. The robot locomotion simulation model has been developed based on the mathematical models and the multibody dynamics simulation tool. Based on the PID algorithm, the simplified locomotion control strategy has been proposed in this study, enabling straight-line motion, turning motion, and nominal path generation of the robot without requiring gait switching. The analytical methods and theoretical models developed in this study are generally applicable to other insect-scale piezoelectric robots.

In contrast to the locomotion control strategy employed by HAMRs [28,29,30], the closed-loop PID controller proposed in this study is oriented toward onboard-deployable control algorithms, featuring advantages such as structural simplicity, low computational requirements, and ease of implementation without relying on precise dynamic models. This makes it particularly well-suited for resource-constrained integrated insect-scale platforms. The control strategy and motion analysis presented in this study provide theoretical foundations for subsequent integrated control development on PLioBot and serve as a reference for hardware refinement.

## 5. Conclusions

This paper investigates the locomotion control of PLioBot, develops mathematical models for its robotic motion processes, and proposes a locomotion control strategy based on a PID-based closed-loop controller. The locomotion of PLioBot is decomposed into a sequence of distinct phases, and the mathematical model governing each phase is formulated accordingly. By integrating the theoretical models with the multibody dynamics tool, we developed the locomotion co-simulation system for the control simulation implementation. The commonly used composite cycloidal trajectories and quintic polynomial bionic trajectories are comparatively analyzed. The motion of the PLioBot under open-loop locomotion is investigated separately for the pace and trot gaits, achieving stable robotic motion. The PID locomotion control strategy is designed to regulate the robot’s motion by adjusting the step lengths of the robot’s left and right legs, eliminating the need to modify the robot’s locomotion gait. The co-simulation control system has been established to implement straight-line locomotion, fixed-angle turning, in-place turning, and nominal path generation locomotion control. The theoretical analysis methodology, robotic simulation approach, and locomotion control strategy proposed in this paper can be extended to other insect-scale micro crawling robots. In future work, we will develop miniaturized high-voltage drive circuits and high-energy-density batteries, fabricate the PLioBot integrated robot, and conduct closed-loop experimental validation.

## Figures and Tables

**Table 1:** The length parameters of the links in parallel-legged mechanism.

Link	Length Parameter
*EF*	*l_n_*
*GH*	*l_n_*
*OF*	*l_m_*
*OH*	*l_m_*
*OA*_1_	*l*_1_
*OA*_2_	*l*_4_
*A*_1_ *P*	*l*_2_
*A*_2_ *P*	*l*_3_
*PD*	*l*_5_

**Table 2:** The differential steering principle of PLioBot.

Step Length	Step Length Difference Δ*S*	Step Length Ratio *k_S_*	Turning Radius *R*	Locomotion
*S_r_* > *S_l_*	Δ*S* > 0	*k_S_* > 1	*R* > 0	Left turn
*S_r_* < *S_l_*	Δ*S* < 0	*k_S_* < 1	*R* < 0	Right turn
*S_r_* = −*S_l_*	Δ*S* ≠ 0 & Δ*S* = 2*S_r_*	*k_S_* = −1	*R* = 0	In-place turn
*S_r_* = *S_l_*	Δ*S* = 0	*k_S_* = 1	*R* = ∞	Straight-line locomotion

**Table 3:** The equivalent stiffness value of the torsional spring at the parallel-legged joints.

Flexible Hinges in Figure 7	E	F	*O*	*A*_1_	*A*_2_	*P*	*H*	*G*
Equivalent stiffness (×10^−7^ N·m/rad)	4.23	4.23	6.77	6.77	6.77	5.64	4.23	4.23

**Table 4:** The density parameters of the linkages and the actuators in the PLioBot model.

Robot Components	Composite Material Linkages	Piezoelectric Actuators
Volume (mm^3^)	9.6	21.6
Weight (mg)	4.8	108
Density (g/cm^3^)	0.5	5

**Table 5:** The contact parameters for the PLioBot feet to the ground in the simulation model.

Parameter Name	Value
Contact stiffness (N·cm^−e^)	1000
Force index	2.1
Contact damping (N·s·cm^−1^)	0.005
Penetration depth (cm)	0.003
Static friction coefficient	1
Kinetic friction coefficient	0.2
Static translational velocity (cm/s)	0.01
Kinetic translational velocity (cm/s)	0.03

## Data Availability

Data is contained within the article.
